# A Roadmap to Perfused Skin: Defining the Next Generation of Research Questions in Cutaneous Tissue Engineering

**DOI:** 10.3390/ijms27125350

**Published:** 2026-06-13

**Authors:** Ahmet Akif Kızılkurtlu, Özgür Yılmaz

**Affiliations:** 1 Department of Biomedical Engineering, Faculty of Engineering and Natural Sciences, Istanbul Atlas University, Istanbul 34403, Turkey; 2 TUBITAK Marmara Research Center, Gebze, Kocaeli 41470, Turkey; 3 Department of Pharmaceutical Chemistry, Faculty of Pharmacy, Istanbul University, Istanbul 34116, Turkey

**Keywords:** perfusion, vascularization, skin tissue engineering, prevascularization, bioprinting, skin-on-chip, translational benchmarking

## Abstract

Cutaneous tissue engineering has advanced from simple coverage substitutes to increasingly complex living constructs, yet the field remains constrained by a decisive problem: timely and durable perfusion. Many engineered skin substitutes can appear vascular in static culture or in small-animal models. However, they still fail when blood flow must be established quickly enough to rescue cells across clinically relevant tissue thickness. Rather than re-catalog platforms already summarized in recent reviews, this critical narrative review reframes the field around perfusion as the master functional endpoint rather than vessel density alone. We analyze the vascularization bottleneck as a sequence, internal network formation, host inosculation, flow initiation, and perfusion stability—and use that sequence to reassess biomaterial design, cell-based strategies, immunomodulation, decellularized matrices, bioprinting, microfluidics, and prevascularization. We intentionally distinguish implantable skin substitutes from perfused in vitro platforms such as skin-on-chip systems, arguing that these are linked but non-interchangeable application spaces with different success criteria. Building on this distinction, we propose a research agenda centered on functional benchmarking of perfusion, spatiotemporal coordination of scaffold dynamics, immune–mural–lymphatic–vascular crosstalk, scalable hierarchical vascular fabrication, and predictive human test platforms. The central argument is that translation will depend not on ever more isolated pro-angiogenic interventions but on integrated systems that survive the ischemic interval, connect rapidly, tolerate blood entry, maintain a workable inflow–outflow balance, and remodel into a stable, skin-specific microvasculature.

## 1. Introduction: From Ready-Made Solutions to Deeper Questions

**Figure 1:**
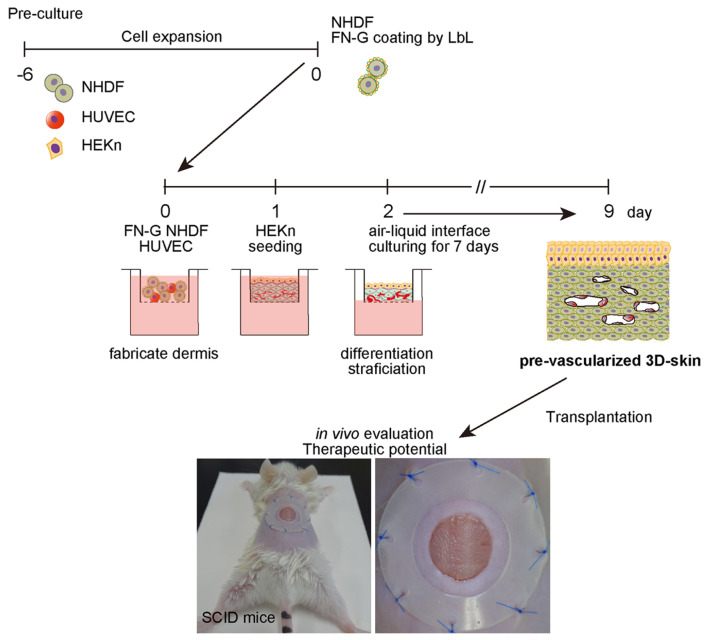
Prevascularization workflow for a scaffold-free dermo-epidermal 3D skin substitute. The scheme links fibroblast/HUVEC co-assembly, keratinocyte seeding, air–liquid interface maturation, and in vivo evaluation. Reprinted with permission from Ref. [30], 2019, Miyazaki et al.

Skin is a unique organ that combines barrier function, sensory perception, and immune surveillance [1,2]. Beyond these roles, skin is now recognized as a neuro-immuno-endocrine organ in which environmental cues are processed by compartmentalized epidermal, dermal, hypodermal, and adnexal regulatory units; through neurotransmitters, hormones, neuropeptides, cytokines, and chemokines, these units link local barrier repair, inflammation, vascular regulation, and tissue remodeling to systemic homeostasis and allostasis [3]. This concept strengthens the rationale for a perfusion-centered roadmap because engineered skin must support not only oxygen and nutrient transport, but also the dynamic neuroimmune and endocrine signaling required for skin-specific homeostatic integration after injury. Its layered architecture is supported by a dense vascular network comprising two plexuses: the superficial sub-papillary plexus with capillaries 10–35 µm in diameter and a deeper cutaneous plexus with vessels 40–50 µm in diameter [2,4]. In healthy human skin, these vessels form hierarchical networks that anastomose vertically and horizontally, ensuring that every cell is within 100–200 µm of a capillary to receive oxygen and nutrients [4,5]. Full-thickness skin injuries damage these capillaries, resulting in ischemia and necrosis within hours unless blood flow is restored [6,7,8,9,10]. Autografts made from split-thickness skin can survive because the thinner dermal component can be sustained by the blood and nutrients that diffuse through the wound bed until inosculation occurs. In contrast, thicker engineered skin products typically die before developing a stable blood supply [11,12,13,14,15,16,17]. There is also a regulatory aspect to the problem: once the dermal microcirculation collapses, oxygen transport decreases, leading to accumulation of metabolic waste products, ongoing inflammation, and disruption of the epidermal/dermal interaction. Stability of the dermal/epidermal interface is especially critical in patients with complex wounds resulting from burns, diabetes, radiation therapy, and those requiring complex reconstructive surgeries [18,19,20,21]; however, the design of engineered skin products for these wounds may vary due to distinct physical requirements. The design parameters that affect the healing process for acute thermal injuries are to cover the wound immediately and to control the risk of infection; the design parameters for the healing of chronic ischemic wounds include the added problem of vascular disease and unresolved inflammation. The design parameters for contouring the new skin, the new product’s handling characteristics, and long-term incorporation into the body are new considerations for patients with complex reconstructive defects. For all types of engineered skin products, the design of the vascular network is not just an important design element; it also serves as the precursor to all other regenerative processes. In other words, unless the vascular network has been established, it will be impossible for the other aspects of the healing process to occur in the proper sequential order; therefore, blood vessels alone may not be sufficient for the healing of damaged skin; this includes the presence of lymphatics, the movement of immune cells through lymphatic vessels, and the regulation of tissue maturation and barrier function by the central nervous system [22,23,24,25]. Therefore, the primary challenge of skin engineering is not only to determine whether vascularity can be created, but to determine whether the localized and durable blood supply can be created in a manner that will permit the layered physiology of normal skin to develop, in addition to allowing for short-term viability of the engineered tissue [5,26,27,28,29]. Initially, tissue engineering focused on developing cell-based scaffolds that could be implanted directly in patients. These initial strategies primarily relied on rapid solutions to augment vascularization, including coating biomaterials with vascular endothelial growth factor (VEGF) or basic fibroblast growth factor (bFGF), seeding endothelial cells (ECs) onto the scaffold, and/or using prevascularized scaffolds. While these techniques improved the vascular network in small-animal models, their clinical application has been limited primarily due to timing issues. Newly formed microvessels grow from an arteriovenous loop at about 5 micrometers/hour; therefore, it may take several weeks for these blood vessels to reach the center of a tissue-engineered construct that is a millimeter thick, leading to irreversible hypoxia-induced injury to embedded cells within several hours to days. Increasingly, the tissue engineering community acknowledges that the temporal element, i.e., when adequate flow (or active perfusion) is sustained, is more critically influential than simply the density of vessels. Figure 1 translates the central vascularization problem into a construct-level workflow: endothelial and stromal co-assembly, epidermal layering, in vitro maturation, and implantation are staged so that the vascular component is present before the host bed must rescue the graft [30].

This review uses the logic of an integrative critical narrative to develop a research agenda that is focused on perfusion as the most constraining variable. For the most part, this review is based on studies involving implantable alternatives to skin; however, we also discuss studies involving perfused in vitro models such as skin-on-chip systems [31,32,33,34,35] and how they are utilized to create discovery platforms, maturation environments, and preclinical evidence of success, rather than as interchangeable clinical products [31,36]. The distinction between these asset types is important because each has different criteria for successful outcomes. To be a successful implant, it must maintain viability upon implantation, inosculate with the host vasculature to sustain blood flow through the implant, and support adequate, consistent wound closure. Accordingly, a microphysiological culture platform must satisfactorily replicate the transport, barrier, and immune functions of the skin, as well as the disease biology of skin diseases, with high reproducibility, so that appropriate preclinical decisions can be made [32,33,34,35]. The purpose of this review is to analyze the similarities and differences between these application areas, define the mechanisms underlying the vascularization bottleneck, establish future research questions to guide testing, facilitate transition to the clinic, and ensure and maintain translational relevance. These include existing strategies for achieving vascularization using skin substitutes; current strategies employed as part of larger pro-angiogenic wound-healing systems; the development of human skin as a vascularized model; and the development of novel technologies for using skin as a bioreactor.

While many reviews have identified the numerous vascularization strategies established to date, the present review has goals that differ from those of these earlier reviews. The focus of this article will be on treating active perfusion as the ultimate goal of all vascularization efforts, separating the vascularization of implantable skin substitutes from their respective in vitro vascular systems, and establishing a linkage between the two based on established vascular principles. Lastly, this article reviews existing strategies, using established benchmarks for both vascularization and translational potential, rather than relying solely on descriptive counts of vascular structures [5,26,27,28,29].

This review is presented as a critical narrative rather than as a systematic review of the literature. The overall focus of this article will be to synthesize information from the published literature on the vascularization of the skin and to generate a framework to establish benchmark goals for meaningfully shortening the time between implantation and the establishment of durable perfusion. To further clarify the indexing of the existing literature, the authors will establish and present a series of interrelated questions about the nature of perfusion and durable perfusion. The four interrelated questions to be discussed are: How do the strategies improve the formation of internal vascular networks? How do they shorten the time to establish perfusion? How do they support the initiation of blood flow? How do they stabilize the flow of blood over time? These methodologies have led to the identification of three general weaknesses in the body of literature reviewed. First, the aesthetically pleasing appearance of vascular networks produced in vitro is often used as a substitute for meaningful perfusion. Second, implantable grafts and skin-on-chip systems are sometimes discussed as though they belonged to the same translational category when, in fact, they solve different problems. Third, evidence levels are frequently conflated, with in vitro promise, rodent efficacy, and clinically relevant translational readiness [5,26,27,28,29] discussed on the same plane. The argument advanced here is that progress will depend less on accumulating isolated pro-angiogenic tricks and more on building integrated systems that can survive implantation, connect rapidly, tolerate shear, resist thrombosis and edema, and mature into skin-specific microvasculature in an indication-appropriate way. 

Several recent reviews have provided valuable catalogs of vascularization strategies for skin tissue engineering, including pro-angiogenic growth-factor delivery, endothelial or stromal cell incorporation, scaffold modification, prevascularization, 3D bioprinting, and microfluidic skin models [26,27,28,29,31,32,33,34,35,37,38,39,40,41,42,43,44,45,46,47,48,49,50,51]. The present review is not intended to duplicate those strategy-by-strategy summaries. Its distinct contribution is to reorganize the field around a perfusion-centered translational framework. In this framework, vascularization is not treated as an endpoint defined by vessel density, endothelial marker expression, or visually attractive network morphology. Instead, vascularization is evaluated as a functional sequence: internal network formation, host inosculation, initiation of blood or medium flow, distributed transport through the construct, leakage and thrombosis control, and long-term patency during remodeling. This sequence allows different technologies to be compared by whether they actually shorten the ischemic interval and support durable perfusion, rather than simply increasing vascular-appearing structures.

This review also introduces a second organizing distinction that is often blurred in the literature: implantable skin substitutes and perfused in vitro skin platforms are related but non-interchangeable translational assets. Implantable constructs must survive implantation, connect to host inflow and outflow, tolerate blood entry, support wound closure, and remain functional in compromised wound beds. By contrast, skin-on-chip and other microphysiological systems are primarily discovery, disease modeling, maturation, and preclinical qualification tools whose success depends on reproducible barrier, vascular, transport, and immune readouts under controlled flow conditions. The new perspective advanced here is therefore not a single new vascularization method, but a decision framework that separates platform purpose, evidence level, and perfusion requirements. Using this framework, biomaterial design, cell-based prevascularization, MVF/SVF strategies, immune–mural regulation, bioprinting, sacrificial-channel systems, microfluidics, and ex vivo flow maturation can be assessed against the same translational questions: Does the strategy build an internal network? Does it accelerate host connection? Does it permit stable blood or medium entry? Does it distribute flow without leakage, shunting, thrombosis, or collapse? And does it provide evidence that can be transferred toward clinically relevant skin repair?

Accordingly, the novelty of this review lies in shifting the discussion from a descriptive inventory of vascularization tools to a functional-perfusion roadmap for engineered skin. This roadmap links mechanism, fabrication strategy, benchmark readouts, evidence hierarchy, and indication-specific translation. It is intended to help the field move from asking whether a construct is vascularized to asking whether it is perfused in a way that is timely, distributed, stable, hemocompatible, and clinically meaningful. Table 1 compares implantable skin substitutes with perfused in vitro platforms.

## 2. Deconstructing the Bottleneck: Angiogenesis, Inosculation, and Perfusion

Effective vascularization of engineered skin requires three distinct but interconnected processes: formation of new vessels within the construct (angiogenesis or vasculogenesis), connection to the host vasculature (inosculation), and establishment of blood flow (perfusion) [52,53,54,55]. Vasculogenesis and angiogenesis refer to the development of new capillary networks, which can occur by two primary routes: (1) the endothelial sprouting of the vascular network from pre-existing vessels (aka, angiogenesis); and (2) the assembly of endothelial cells and perivascular cells into lumenized capillary networks (i.e., vasculogenesis). The process of inosculation entails a direct connection between engineered capillaries and host vessels, thereby providing a conduit for blood delivery to tissues [36,56,57]. Perfusion refers to the physiological maintenance of blood flow through the capillary network at physiologic pressures. Any inappropriate or inefficient performance of one of these three processes (angiogenesis, vasculogenesis, or inosculation) can ultimately lead to ischemia or necrosis. Thus, the three-step breakdown is not simply a semantic way of describing success; it clearly defines the parameters needed for success to be considered achieved. Specifically, an engineered construct may include several endothelial cords, but if those cords do not form stable lumens, do not connect to the host, or thrombose upon the first exposure to blood flow, then it cannot be considered successful. Conversely, an engineered construct may promote significant infiltration of host tissue at its periphery. Still, if there is no perfusion through the inner portion of the engineered construct, the construct will present an attractive histological appearance. Still, it will have made little, if any, functional contribution to the tissue’s recovery. Therefore, additional studies should focus on vascularization, using staged endpoints that examine multiple aspects of vascularization across a continuum of staged performance domains [5,58,59,60,61]. The following are examples of staged performance domains: the general morphology of the network; whether the endothelial cords are continuous and stable; whether the endothelial cords have adequate coverage of mural cells; and the permeability of the networks. To further delineate the vascularization bottleneck into functional domains, four distinct domains of failure can explain graft failure: inadequate internal network formation, failure to connect to host vessels, poor balance between inflow and outflow, and failure of newly formed connections to withstand blood flow.

**Figure 2:**
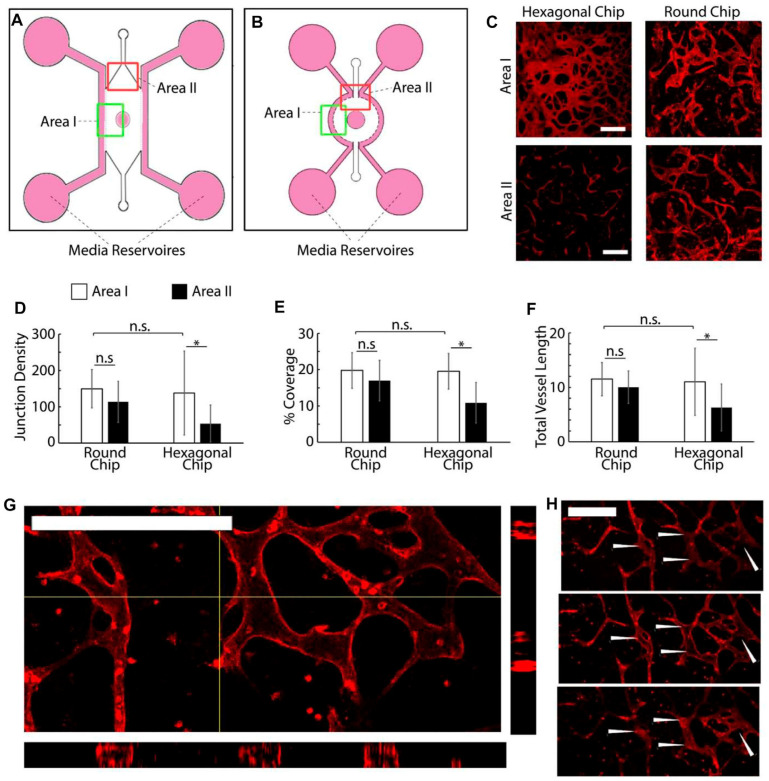
Microfluidic chip geometries and endothelial network organization in a vascularized skin-on-chip platform. The panels compare hexagonal and round chip layouts, microvascular morphology, network metrics, and lumen-like structures. Green squares refer to a 380 μm long and 90 μm wide area, and red squares refer to a 3 mm diameter circular central well open to the air. * *p* < 0.05; n.s., non-significant, *p* > 0.05; error bars are standard errors; n = 3. Scale bars: 200 μm. (**A**,**B**) Schematic representation of the two chip designs investigated. (**C**) Examples of microvascular networks formed in different areas of the central compartment of hexagonal and round chips. Scale bars: 200 μm. (**D**−**F**) Quantification of the morphology of the corresponding networks. Junction density and total vessel length are expressed in arbitrary units normalized by the section area. (**G**–**H**) Confocal images of networks formed in round chips displaying luminated structures. Reprinted with permission from Ref. [31], 2022, Jones et al.

Figure 2 illustrates why morphology alone is insufficient: chip geometry, junction density, luminal continuity, and orthogonal imaging all influence whether an apparent network can be interpreted as a perfusable system rather than only a vascular-looking pattern [31,57,59,62,63].

### 2.1. Angiogenesis Versus Vasculogenesis

To typically trigger angiogenesis in vivo, scaffolds are used to deliver genes and/or growth factors (VEGF, bFGF, and PDGF) [64,65,66,67,68]. Hydrogels containing VEGF have shown accelerated wound healing through improved vessel density and decreased fibrotic formation, whereas hydrogels delivered with bFGF enhanced the potential for cell replication and re-epithelialization [67,68,69,70]. Matrices containing VEGF plasmid DNA will ultimately lead to the formation of mature blood vessels and increase the tensile strength of the newly formed tissue [69,70,71]. The main challenge is to ensure that the new host sprouts arrive before irreversible changes occur due to central ischemic injury. It has been found that the intrinsic growth rate of sprouting is 5 µm/hour, with host endothelial competence, matrix accessibility, and inflammatory context substantially impacting it. As a result, pro-angiogenic strategies are particularly vulnerable to diabetes, aging, irradiation, and vascular disease. Furthermore, it is also important to consider how the multiple cues will be presented to the host. In natural healing processes, VEGF and other similar angiogenic factors are bound to and released from a spatially heterogeneous matrix, whereas uncontrolled delivery of biomaterials may result in increased density of peripheral vessels but may still produce fragile vessels that are hyperpermeable and poorly stabilized in deeper layers of the matrix [64,65,66,67,68,69,70,71]. For the thicker constructs, there are specific design questions that relate to how it is possible to create ’context-specific angiogenesis’, e.g., how to adjust timing and dose, how to provide matrix anchorage and how to allow for the sequential signaling to occur to enable early sprouting to be transitioned into mural recruitment, basement-membrane deposition and pruning of non-viable vessels, rather than the maintenance of immature vessels.

**Figure 3:**
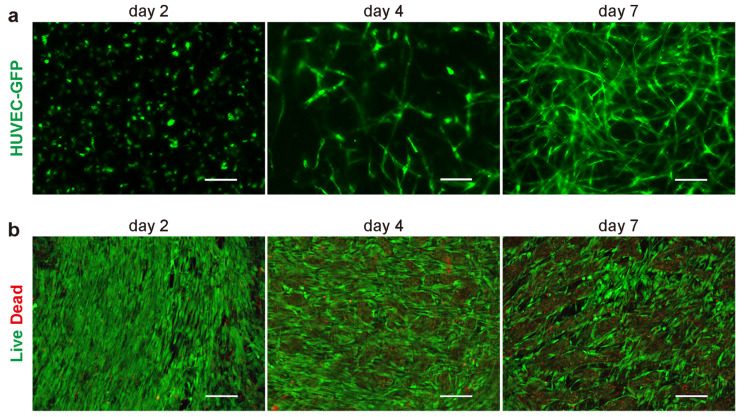
Time-dependent formation of endothelial vessel-like networks in dermo-epidermal 3D skin substitutes during air–liquid interface culture, with parallel live/dead viability assessment. (**a**,**b**) GFP-expressing HUVEC or HUVEC (1 × 10^5^ cells/insert) mixed with FN-G-coated NHDF (1 × 10^7^ cells) were cocultured, subsequently covered with HEKn (1 × 10^6^ cells), then cultured for up to an additional 7 days. (**a**) The dynamics of vessel-like formation in organotypic cocultures. Representative images of vascular network formation within 3D skin after air–liquid interface cultivation. (**b**) The viability of the cocultured cells in the 3D skin as assessed by LIVE/DEAD assay. Reprinted with permission from Ref. [30], 2019, Miyazaki et al.

Figure 3 illustrates the formation of the vasculogenic network over time. The same construct that initially contains dispersed endothelial cells can progressively develop elongated, connected structures while viability remains broadly preserved during early culture [30].

**Figure 4:**
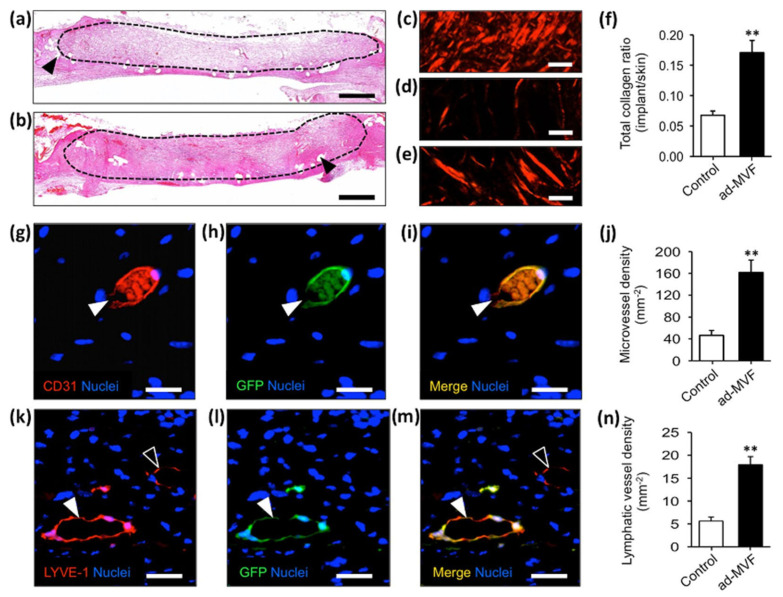
Histological and immunohistochemical evidence of microvessel and lymphatic vessel formation in ad-MVF-prevascularized dermal substitutes. (**a**,**b**) HE-stained sections of non-seeded (**a**) and prevascularized (**b**) implants. Broken line = implant border, arrowheads = mesh fibers. (**c**–**e**) Polarized light microscopy of Sirius red-stained sections of normal skin (**c**), non-seeded (**d**) and prevascularized (**e**) Integra. (**f**) Quantification of total collagen ratio (implant/skin). Mean ± SEM, n = 8, ** *p* < 0.001 vs. non-seeded control. (**g**–**i**,**k**–**m**) Immunohistochemical staining of microvessels (**g**–**i**, white arrowhead = CD31^+^/GFP^+^ microvessel) and lymphatic vessels (**k**–**m**, white arrowhead = LYVE-1+/GFP+ lymphatic vessel, empty arrowhead = LYVE-1+/GFP− lymphatic vessel) within prevascularized Integra 21 days after implantation. (**j**,**n**) Quantification of microvessel density (mm^−2^) and lymphatic vessel density (mm^−2^). Mean ± SEM, n = 8, ** *p* < 0.001 vs. non-seeded control. Scale bars: (**a**,**b**) = 800 µm, (**c**–**e**) = 20 µm, (**g**–**i**) = 20 µm, (**k**–**m**) = 30 µm. Reprinted with permission from Ref. [74], 2018, Frueh et al.

Vasculogenesis, or the assembly of dispersed vascular progenitor populations into organized vascular structures, could provide another approach to forming vascular networks in vivo [52,53,54,55,56]. Culturing endothelial cells (ECs) with perivascular cells or stroma can lead to spontaneous network formation. Microvascular fragments (MVFs) obtained from adipose tissue consist of intact arteriolar, capillary, and venular segments that are up to 150 µm long [72,73,74]. When implanted, MVFs rapidly assemble into perfused networks and secrete pro-angiogenic factors. In experimental models of ischemic hindlimb, MVFs exhibited superior blood flow and angiogenesis compared with mesenchymal stem cells (MSCs) or stromal vascular fractions (SVFs) [75,76]. MVF-seeded skin grafts are promoted for their improved integration into host tissue and promote inosculation with host vessels. MVFs retain small segments of lumenized vessels and associated supportive tissue and basement membrane, thereby, to the extent possible, reducing the need to rearrange the cells delivered in a cell suspension at the time of implant. This may shorten the time to connect with the host [72,73,74,75,76]. However, the manufacture of vasculogenic systems is complex and should therefore be performed with greater rigor than conventional vascular implants. MVF yield is affected by tissue source, digestion protocol, and donor variability; therefore, handling time affects MVF viability, and methods for short-term storage, quality control, and scaling up the overall manufacturing process remain undefined. Therefore, there currently exists a framework for practical decision-making: host-motivated angiogenesis is simpler and potentially less expensive than MVF systems, but is slower to achieve and more dependent on the host. In contrast, prevascularized or MVF-based systems are expected to form inosculation more quickly but require greater effort to develop and control throughout the manufacturing and release of the final product. Figure 4 highlights the distinctive logic of microvascular fragments: the implanted material contains pre-existing multicellular vascular units that can contribute not only to microvessel density but also to the formation of lymphatic-like structures and matrix integration [73,74].

### 2.2. Inosculation and Perfusion

**Figure 5:**
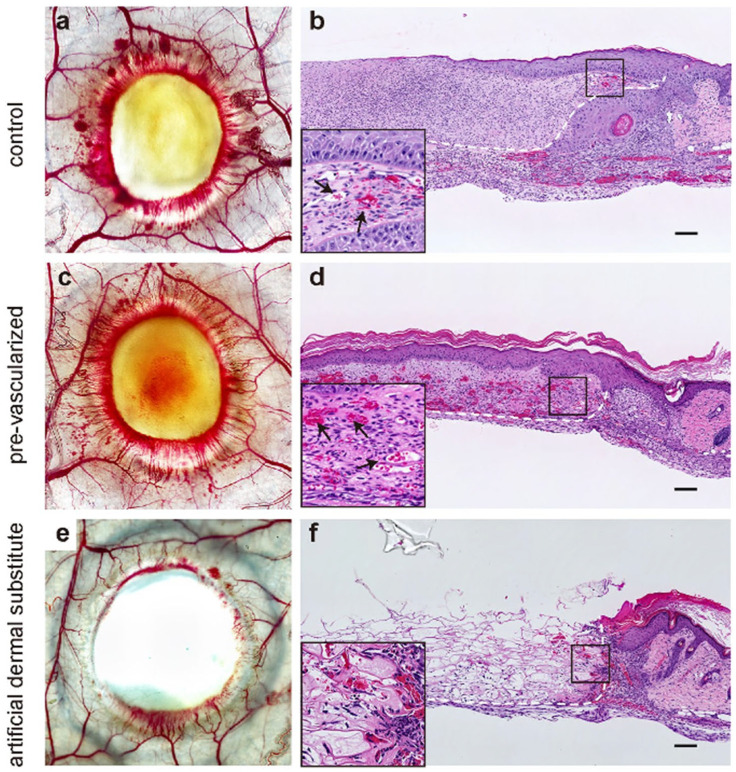
Rapid induction of functional vasculature in prevascularized 3D skin substitutes in vivo. Transillumination and histology show early blood-perfused vessels in the prevascularized graft group. (**a**–**f**) Transillumination microscopy (**a**,**c**,**e**) and hematoxylin and eosin-stained sections (**b**,**d**,**f**) of each substitute 7 days after grafting. Reprinted with permission from Ref. [30], 2019, Miyazaki et al.

Inosculation is the fusion of engineered and host vessels [36]. Classical skin grafts rely on inosculation to reestablish circulation; host blood appears in graft capillaries within four days [36,58]. In contrast, thick dermal substitutes like Integra require neovascularization through the scaffold, a process that can take 2–5 weeks. Engineered skin must therefore present a microvascular environment that can connect to host vessels before the diffusion window closes. Studies using endothelialized skin constructs have demonstrated rapid inosculation and perfusion within days, highlighting the importance of pre-established networks and endothelial lining [30,36,56,57,58,59,62,63,77,78]. Stable perfusion, however, begins rather than ends at anastomosis. Once blood enters a preformed network, endothelial cells are exposed to shear stress, cyclic loading, plasma proteins, platelets, and inflammatory mediators that can rapidly alter barrier behavior and gene expression [60,61,79,80]. Immature networks may leak, detach, thrombose, or shunt flow through a few low-resistance channels. Perfusion, therefore, depends on wall maturation, hemocompatibility, hierarchical branching, and workable inflow and venous outflow. Clinical grafts are not only an issue of capillary fusion; they also rely on microvascular beds that connect to the recipient’s macrovascular system. There are situations in which some of these connections require supportive surgical techniques or engineered channels to create a more direct connection between the microvasculature and the recipient’s macrovascular system. Thus, the goal of translation is to facilitate rapid connection between the microvasculature and macrovasculature, resulting in functional integration with the host’s physiologic blood flow [5,58,59]. Figure 5 emphasizes the distinction between vascular presence and functional blood entry. The key observation is not only that vessels are visible around the graft, but also that blood-perfused vessels are detectable within the prevascularized substitute during the early post-grafting window [30].

### 2.3. The Time Race

**Figure 6:**
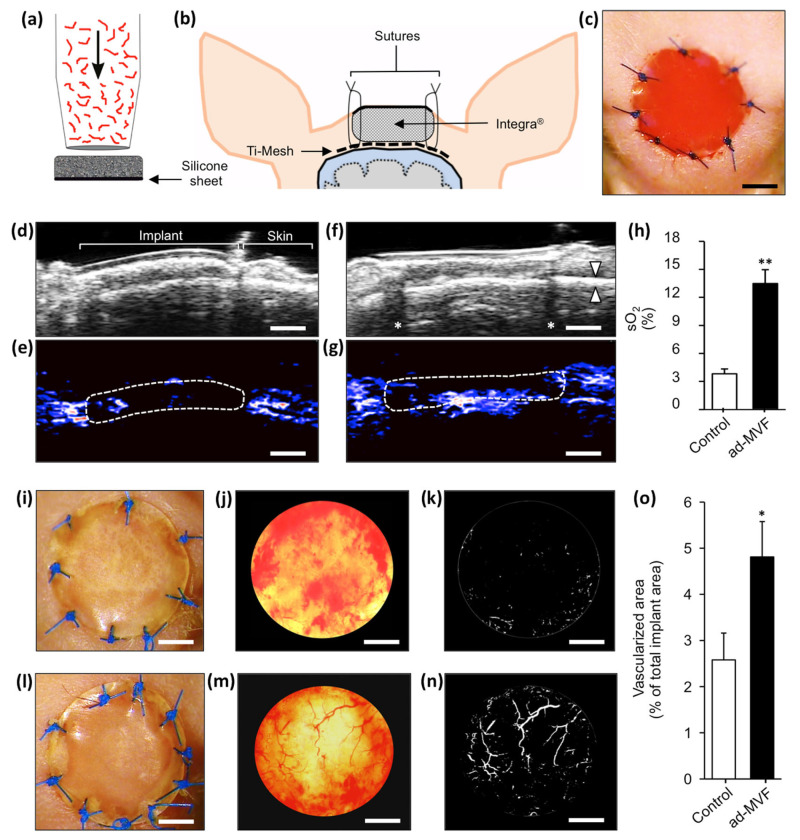
Animal model, photo-acoustic oxygenation imaging, and transillumination stereomicroscopy for non-seeded and ad-MVF-prevascularized Integra matrices. The figure connects prevascularization to increased oxygenation and to the vascularized implant area. (**a**–**c**) After the seeding process (**a**), the matrices were implanted into full-thickness skin defects on the skull of CD1 nu/nu mice (**b**,**c**). (**d**–**g**) B-mode ultrasound (**d**,**f**) and OxyHemo-mode photo-acoustic imaging (**e**,**g**) of non-seeded (**d**,**e**) and prevascularized (**f**,**g**) Integra 21 days after implantation. Red areas = high oxygenation, blue areas = low oxygenation, arrowheads in f = frontal calvaria, asterisks in f = dorsal acoustic attenuation (sutures), broken line in (**e**,**g**) = implants. (**h**) Quantification of sO2 (%). Mean ± SEM, n = 8, ** *p* < 0.001 vs. non-seeded control. (**i**–**n**) Epi-illumination (**i**,**l**) and trans-illumination (**j**,**m**) microscopy with digital segmentation images (**k**,**n**) of non-seeded (**i**–**k**) and prevascularized (**l**–**n**) Integra. (**o**) Quantification of vascularized area (% of total implant area). Mean ± SEM, n = 8, * *p* < 0.05 vs. non-seeded control. Scale bars: (**c**) = 2.5 mm, (**d**–**g**) = 1.8 mm, (**i**–**n**) = 2 mm. Reprinted with permission from Ref. [74], 2018, Frueh et al.

The diffusion limit of O2 diffusion is between 100 and 200 µm, meaning a cell at a depth greater than this distance must receive blood within a few days of implantation to survive [5,56,58]. Angiogenic sprouting, however, extends only a few micrometers per hour, resulting in a gap between the time required to implant and perfuse a functional network of vessels and the time available to the host [5,26,27,28,29,52,53,54,55]. Therefore, engineered tissues may have to be implanted with functional, perfusable vessels, or the host will have to inosculate (into the engineered network) and perfuse it quickly. For this to occur, the scaffold design, cell biology, immune response, and biomechanics must be in perfect harmony. Additionally, this temporal mismatch can prompt us to reconsider many of the design choices we currently use in the field. For example, although a higher cell density within a construct may lead to a faster rate of matrix production and/or increased paracrine signaling, which would be beneficial for the construct, it also increases the construct’s metabolic demands. It decreases the duration that it can survive without having blood flow. Likewise, thicker analogs of human skin may provide a more appropriate design for the mechanics of the dermis; however, each additional 100 µm will further exacerbate the oxygen-delivery issue unless convective transport is incorporated into the construct. If the construct is placed in a wound environment, the survival window will be further reduced due to edema, acidosis, bacterial infection, and the host’s inability to provide perfusion [79,80]. So, how long does it take for a stable blood supply to be available for the skin graft? Time until stable perfusion will likely be the most informative systems-level metric available to the field for the development of future generations of engineered skin substitutes. If the metric can be improved by one or two days, it may dramatically change the survival of a skin graft. Therefore, we need to consider not just how to obtain the blood vessels in the engineered tissues, but rather how to provide a means of extending the time the construct can survive without a functional blood supply (i.e., through transport channels, the ability to reduce metabolic load during initial stages of implantation, the presence of O2-releasing systems, the creation of pre-existing lumens, or surgical techniques that more directly couple the construct to the host blood supply immediately after implantation). All in all, every successful engineered tissue construct must resolve an oxygen-delivery issue before a tissue-organization solution can be achieved. The principal strategies that will extend the construct’s survival time and shorten the time to establish blood flow are summarized in Table 2. Figure 6 links the time-race concept to measurable oxygenation and vascularized area. It shows how prevascularization can be evaluated by dynamic and macroscopic readouts rather than by endpoint histology alone [74].

### 2.4. What Should Count as Functional Perfusion?

Because functional perfusion is the central endpoint of this review, it should be defined by measurable performance rather than by vascular appearance alone. In engineered skin, CD31-positive structures, vessel density, or visually attractive endothelial networks should be regarded as evidence of vascular organization, but not sufficient evidence of healthy perfusion. A construct should be considered “perfusable” only when it contains patent lumens that can be connected to an imposed flow circuit or host vasculature. It should be considered “functionally perfused” only when flow enters the network, traverses a meaningful fraction of the construct, delivers oxygen and nutrients across the intended thickness, remains stable without catastrophic leakage or thrombosis, and produces a downstream tissue-level benefit such as improved viability, graft take, barrier recovery, or wound closure [5,26,27,28,29,36,56,57,58,59,60,61,62,63,77,78].

For this reason, effective perfusion should be evaluated as a tiered claim rather than as a binary yes/no outcome. The first tier is anatomical readiness: continuous endothelialized lumens, junctional integrity, and, where possible, mural-cell or basement-membrane support. The second tier is flow access and transit: evidence that red blood cells, fluorescent tracers, microspheres, culture medium, or angiographic contrast can enter and move through the construct rather than remaining at the periphery. The third tier is the transport function: reduced hypoxic zones, improved oxygen tension, preserved metabolic activity, and improved cell viability across the full construct thickness. The fourth tier is vascular stability: controlled permeability, resistance to thrombosis, maintenance of patency, balanced inflow and outflow, and avoidance of shunting through only a few low-resistance channels. The final tier is graft-level functional rescue: survival of the engineered skin, stable wound integration, epidermal barrier recovery, and durable closure in an indication-relevant model [49,50,57,58,59,60,61,62,63,72,73,74,75,76,77,78,79,80,81,82].

No single universal numerical threshold can define effective perfusion across all skin constructs because construct thickness, vascular geometry, channel diameter, cell density, biomaterial stiffness, host bed condition, and intended clinical indication differ substantially. However, each study should predefine its intended perfusion claim, the time window in which perfusion must occur, the construct thickness over which oxygen rescue is expected, and the minimum evidence package needed to support that claim. For implantable skin substitutes, the most informative primary benchmark is time to distributed, stable perfusion after implantation. For skin-on-chip and other in vitro platforms, the most informative benchmarks are controlled flow rate, endothelial barrier behavior, reproducible oxygen/nutrient transport, and assay repeatability. Table 3 summarizes a practical benchmark set for evaluating the standard perfusion in engineered skin.

## 3. Scrutinizing Current Strategies to Formulate Future Questions

There are currently several approaches to vascularizing dermal substitutes, which can be categorized into three main classes: biomaterial-based approaches, cellular/biochemical crosstalk, and advanced bioprinting/3D bioprinting technologies [68,69,70,71,72,73,74,75,76,77,78,79,80,81,82,83,84,85,86,87,88,89,90,91,92,93,94,95,96,97,98,99,100,101,102,103,104,105]. While each of these approaches has demonstrated potential to facilitate the vascularization of dermal grafts, it remains unclear whether either will ultimately be successful in supporting the complete continuum from scaffold formation through sustained delivery of blood flow to the recipient tissue. There are substantial interactions between these three classes of approaches. The stiffness, topography, and rate of material degradation govern cellular compliance, whereas the scaffold’s ability to induce cellular crosstalk determines whether the cellular environment produces vascularized or fibrous tissue. Finally, bioprinting/3D bioprinting technologies [37,38,39,40,41,42,43,44,45,46,47,83,84,85,86,87,88,89,90,91,92,93,94,95,96,97,98,99,100,101,102,103,104,105,106,107,108,109] enable the spatial precision with which biomaterials and cellular cues can be configured both geometrically and biologically. This article’s examination of these strategies is conducted in response to four questions, namely: Will they accelerate the development of internal vascular architecture? Will they shorten the time between the initiation of the vascular connection and successful perfusion? Will they enhance the inherent ability of the vascular structure to maintain blood flow and volume? Will they be reproducibly manufacturable for the intended use? The fifth question concerns the ability of each of these strategies to be applied consistently at each stage without compromising hemocompatibility, fluid transport, or the vascular structure’s capacity to provide microenvironmental support to the surrounding tissue. In evaluating the differences and similarities between the implantable constructs (i.e., vascularized dermis) and the perfused in vitro model systems (i.e., tissue- or cell-derived microvascular grafts), the evaluation criteria for each type of graft will differ. The evaluation of the implantable constructs will focus on surgical handling and wound healing, while the evaluation of the in vitro systems will focus on their reproducibility and predictive power. However, while these two types of grafts should be compared, they should not be treated as being interchangeable [48,49,50]. A detailed summary of the major classes of vascularization strategies and their progression through each identified stage of perfusion will be provided in Table 4.

### 3.1. Biomaterial-Driven Approaches

#### 3.1.1. Topography, Porosity, and Degradation

While scaffold architecture has a large impact on vascular ingrowth, there is currently no evidence in the literature indicating a single universal “best pore size” for scaffolds [83,84,85,86]. Interconnecting porosity allows for endothelial infiltration and nutrient diffusion, but how cells attach, migrate, and remodel scaffolds is influenced by a combination of pore size, pore shape, pore tortuosity, and interconnectivity. Reported ranges of pore sizes provide valuable information—smaller pore sizes favor keratinocyte attachment and organization at the surface, while larger interconnected pore sizes enhance fibroblast migration and vascular growth into the deeper layer for skin—but ultimately, they are more of a guideline than a strictly transportable design rule as they vary depending on the material’s composition and the anticipated end point of the constructed tissue. For the skin, the most useful principle is the hierarchical scaffold architecture, wherein surface microfeatures enable epidermal growth and dermal compartments allow fluid transport, capillary invasion, and ultimately the joining of the epidermis with the dermis [83,84,85,86,87,88,89,90,91,92,93,94,95,96]. Future work should focus on both determining the optimal pore size to increase vessel counts and investigating the types of architectures that best support and coordinate attachment, invasiveness, drainage, mechanical properties, and multilayered tissue organization. 

**Figure 7:**
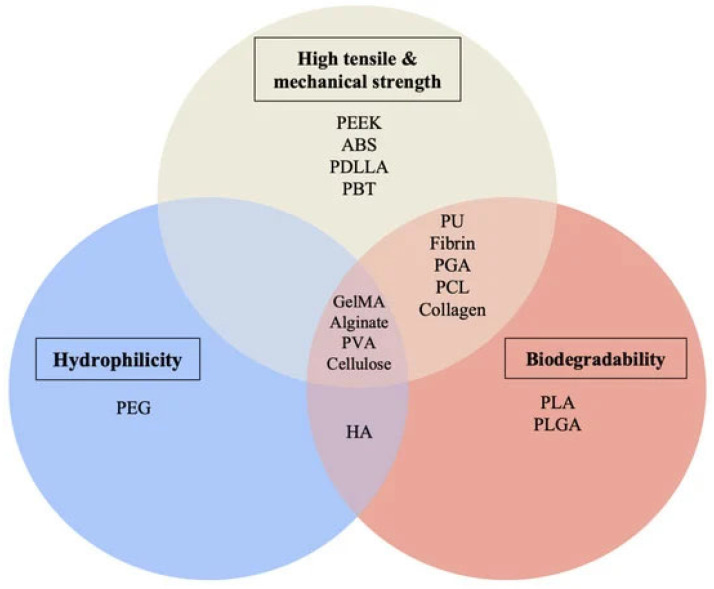
Summary of natural and synthetic polymer properties relevant to scaffold and bioink selection, including hydrophilicity, biodegradability, and mechanical strength. Reprinted with permission from Ref. [51], 2023, Mir et al.

The clinical failure of a scaffold that facilitates the ingrowth of vascularity can occur in thick grafts if it does not allow drainage of exudate or the free flow of venous and lymphatic fluid to the graft’s skin surface. Therefore, a consideration of scaffold architecture should be viewed as a finding of low-to-net utility, but as a guide to optimizing scaffold opening to support vascular growth, and ultimately the mechanical resistance to wound contraction and the capability to sustain the creation of functioning vascular and epidermal structures. Additionally, to effectively synchronize the rate of scaffold degradation with the rate of tissue regeneration through the scaffold, there needs to be a strong correlation between tissue repair and scaffold degradation, which can be achieved using smart polymers that respond to specific enzymes or mechanical stresses. The use of methacrylated hyaluronic acid (HA) hydrogels loaded with mesenchymal stem cells (MSCs) has been shown to decrease post-surgical inflammation and promote the rate of angiogenesis [87,88,89,90,91,92,93,94,95,96], whereas silk fibroin/gelatin constructs formed using digital light processing have been used to create multilayered models of the dermis and epidermis and degrade at a rate that matches the creation of new tissue. However, degradation is not simply a matter of half-life; a variety of factors affect a scaffold’s degradation after placement, including the material’s spatial arrangement, degradation products, and mechanical properties. For example, softening a scaffold will result in a loss of geometric form before the vessels stabilize. Alternatively, if the scaffold is degraded by cells responding to degradation products, cell-specific degradation will enable the formation of access routes to a capillary network where they are most needed. Furthermore, the presence of degradation products within the scaffold can alter its pH, thereby affecting the scaffold’s immune properties, and both the scaffold and the tissue formed will need to maintain a degree of permeability to allow budding of new capillaries while resisting fibroblast migration. Acute burns will generally require rapid opening and coverage of the wound. In contrast, time-dependent chronic wounds typically require longer material support to promote drainage, control inflammation, and replace the base matrix. The focal point for future research is how to program the spatial and temporal degradability of scaffolds so that vascular ingrowth, tissue maturation, and dermal remodeling occur cooperatively rather than competitively. In terms of evidence to support the ability to control the level of vascular ingrowth in vitro and in rodent models [83,84,85,86,87,88,89,90,91,92,93,94,95,96], the strongest data will be based on determining the ability of the scaffold to permit the elevation of tissue using controlled perfusion and drainage, with even the most basic "tunable" flow-competence and drainage data still being limited. Figure 7 summarizes why scaffold material choice cannot be reduced to a single pore-size rule. Hydrophilicity, biodegradability, and mechanical strength must be balanced because each property affects cell invasion, channel stability, degradation timing, and surgical handling.

#### 3.1.2. Bioactive and Gene-Activated Scaffolds

A traditional approach to loading scaffolds with growth factors is to use hydrogel carriers containing an appropriate combination of VEGF and bFGF, thereby promoting angiogenesis and enhancing wound healing [64,65,66,67,68,69,70,71]; however, the use of such exogenous agents may lead to abnormal angiogenesis due to diffusion or other mechanisms. Plasmid DNA containing pro-angiogenic factor-coding genes can also be delivered via gene-activated scaffolds, thereby enabling localized production of these factors [110,111,112]. A recent study using a non-viral GET peptide-based delivery system to create a VEGF-gene-activated scaffold demonstrated significant reductions in cytotoxicity and enhanced VEGF production in human dermal fibroblasts as compared to commercially available vectors; in vitro studies revealed that these VEGF-gene-activated scaffolds stimulated cross-talk between fibroblasts and endothelial cells (ECs) as well as supporting neurite growth in vitro [112,113]. Therefore, gene delivery may enhance both neurogenesis and angiogenesis. Nonetheless, there are differences in how proteins and gene products can be delivered. While protein delivery is considered more straightforward from a pharmacological standpoint, it has the disadvantage of rapid burst release and short half-lives in vivo. In contrast, the additional complexities associated with delivering genetic material (such as the potential for decreased transfection efficiency, extended periods of expression, the possibility of causing inflammation due to unintentional activation of the immune response, stability issues, and the increased regulatory burden placed on product approval) create unique challenges [110,111,112,113,114,115,116,117].

The next generation of bioactive scaffolds will likely depend on signal logic rather than payload alone: what is released, where, for how long, and in what sequence relative to mural recruitment, barrier tightening, inflammation control, and the onset of blood entry. These approaches may be especially attractive in acute settings where endogenous responsiveness is still recoverable, but they remain less predictable in chronically inflamed or vasculopathic wound beds. Most evidence for gene-activated approaches remains in early preclinical studies, with limited standardized data on flow tolerance, thrombogenicity, or manufacturability.

#### 3.1.3. Decellularized and Micronized ECM

Decellularized extracellular matrix (dECM) retains native proteins and bioactive molecules while removing most immunogenic components, providing a biomimetic environment. dECM has been shown to modulate macrophage polarization from a pro-inflammatory M1 phenotype to an anti-inflammatory M2 phenotype, enhancing angiogenesis, fibroblast activation, and ECM synthesis [118,119]. Micronized acellular dermal matrix (mADM) produced via supercritical carbon dioxide decellularization retains growth-factors and promotes angiogenesis [120]. mADM-treated human umbilical vein ECs exhibit upregulation of angiogenesis-related genes and activation of AKT/ERK pathways [120]; injection of mADM induces neovascularization with CD31-positive vessels in vivo. This research demonstrates that ECM-derived materials may provide both structural support and immunomodulatory effects. Although it is common to classify implant scaffolds, print-on-demand dECM bioinks, and inject dECM formulations containing micronized ECM under a single heading, doing so fails to recognize the unique mechanical, sterility, storage, and batch-consistency requirements imposed on each class. A variety of factors, including source tissue, decellularization chemistry, sterilization, and post-processing, affect which instructive cues remain in ECM-derived materials; therefore, the main question is not if these cells have been completely removed, but which matrix signaling molecules, growth factor binding sites, and immunomodulatory properties can be consistently processed and retained following processing [118,119,120,121,122,123,124]. There is a significant biological rationale for these materials, yet confidence in their translation remains limited due to batch-to-batch variability, insufficient release testing, and limited comparative data. Given these variables, the ECM-derived materials should be conceptualized as interwoven design factors rather than being treated as stand-alone formulations (Table 5).

### 3.2. Biological and Cellular Crosstalk

Given that perfusion, or “blood flow” to tissues, is a time-based phenomenon [60,61], the terms “biological crosstalk” and “biological crosstalk actors” refer to cellular interactions and their timing as a sequence of events. The establishment of an early immune response and the delivery of trophic factors to support endothelial cell migration and growth into a new blood vessel network, or “sprouting,” require an inflammatory reaction and several growth factors [97,98,99,100]. The ability of endothelial cells to inosculate with other endothelial cells to form new blood vessel structures through overlapping cell–cell and cell–matrix interfaces is dependent on endothelial cell preparation, the availability of a suitable extracellular matrix for reconstruction of the basement membrane, and the overall resolution of the immune response to injury will be stable and sustained. As such, when evaluating the impact of a given cell type or signal on the development of an angiogenic response, it is important to consider not only the time at which the cell type or signal has produced an effect, but also the manner in which it produces that effect on a specific angiogenic pathway and how it must ultimately be translated into a functioning capillary bed via a perfusion system (Table 6).

#### 3.2.1. Growth Factors and Cell Therapies

**Figure 8:**
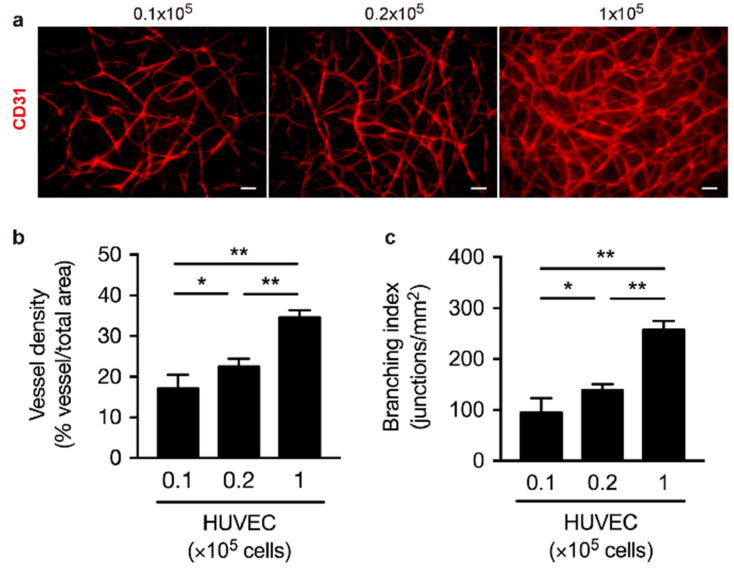
Effect of endothelial cell seeding density on interconnected CD31-positive network formation, vessel density, and branching index in 3D skin substitutes. (**a**–**c**) HUVEC were seeded at 0.1 to 1 × 10^5^ cells per culture insert; FN-G-coated NHDF content (1 × 10^7^ cells) remained constant. FN-G-coated NHDF and HUVEC were cocultured at 1000:1, 500:1, or 100:1, and then with HEKn (1 × 10^6^ cells). (**a**) Whole-mount CD31 immunofluorescence analysis revealed that HUVEC developed branching vessels within the dermal layer. The data are representative of 2 independent experiments (n = 6/condition). (**b**,**c**) Quantification of vascular structures to determine the vessel density (**b**) and branching index (branching points/unit area) (**c**). The data are the mean ± SD (n = 6). * *p* < 0.05 and ** *p* < 0.01 determined by one-way ANOVA with Tukey’s multiple comparison post hoc test. Reprinted with permission from Ref. [30], 2019, Miyazaki et al.

Although vascular endothelial growth factor (VEGF), basic fibroblast growth factor (bFGF), and platelet-derived growth factor (PDGF) are considered pro-angiogenic growth factors, the use of transient methods for delivering the proteins does not produce the same adaptive behavior that is observed in vivo due to the combination of other cell types with endothelial cells [64,65,66,67,68,69,70,71,97,98,99,100]. Endothelial cells need support from pericytes and fibroblasts to achieve stability as nascent vascular structures form [101,102,103,104,105]. MSCs secrete a host of pro-angiogenic cytokines (IL-6, IL-8, IL-10, VEGF) and improve wound healing, yet their engraftment is poor, and efficacy varies [106,107,108,109,125,126]. This has increased interest in secretome- and extracellular-vesicle-based strategies that may capture part of the trophic benefit while easing manufacturing and storage. By contrast, MVFs assemble into networks quickly and promote inosculation [72,73,74,75,76]. Prevascularized 3D skin constructs seeded with MVFs increased hypoxia-inducible factor-1a expression and vascular area in wounds, leading to healing dynamics comparable to split-thickness grafts. The practical question is therefore comparative, not ideological: when is adaptive cellular behavior indispensable, and when can a more standardized acellular or semi-cellular system deliver equivalent benefit? The answer will depend on whether the therapeutic goal is early rescue, durable remodeling, or both, and on whether the target indication is an acute burn, a chronic ischemic wound, or a reconstructive defect with greater tolerance for manufacturing complexity. Evidence is currently stronger for trophic benefit and early preclinical efficacy than for durable engraftment or head-to-head superiority over better-standardized acellular alternatives [114,115,116,117,118,119,120,121,122,123,124]. Figure 8 illustrates that endothelial–stromal ratios are not a minor culture detail; they directly alter vascular density and branching. This supports the argument that cellular crosstalk must be quantitatively benchmarked rather than inferred from the presence of endothelial cells alone [30].

#### 3.2.2. Immune Modulation

The immune system orchestrates tissue regeneration. Macrophages transition from a pro-inflammatory M1 phenotype to a pro-healing M2 phenotype during wound healing [97,98,99,100]. Contrary to the traditional view, primary human M1 macrophages secrete high levels of VEGF, while M2a macrophages secrete PDGF-BB to recruit pericytes, and M2c macrophages secrete matrix metalloproteinase-9 for vascular remodeling. Both M1 and M2 phenotypes are needed; early M1-associated signals help initiate sprouting, whereas later M2-dominant programs support vessel maturation. Decellularized ECM materials can modulate this switch to enhance angiogenesis, but the mechanisms remain incompletely defined. The objective of designing biomaterials should not be to suppress inflammation through the use of an anti-inflammatory drug, but rather, to provide the ability to control the immune response temporally. When designing a biomaterial, macrophage recruitment and phenotype can be strongly influenced by many of the properties of the material itself, including pore architecture, stiffness, surface chemical composition, degradation products, and ligands embedded in the matrix. In addition to the need to develop better means for predicting the ability of biomaterials to provide temporal control over the immune response to promote healing, the field would benefit from additional, testable hypotheses regarding the identification of specific properties of biomaterials that can induce a productive early inflammatory window and trigger resolution of inflammation, as well as how those properties should be optimized to synchronize with the maturation of endothelial barriers. This challenge is particularly relevant to wound healing in patients with diabetes, infections, and radiation exposure, where immune dysregulation and vascular insufficiency interact [18,19,20,21].

Pericytes also play a critical role: they detach from existing vessels to form tunnels for endothelial sprouts and guide new vessel formation [101,102,103,104,105]. However, they also express CXCL10 and CXCL11 to limit endothelial migration and promote vascular regression, indicating a dual role in angiogenesis. PDGFR-b signaling is essential for pericyte recruitment [104,105]; under hypoxia, pericytes secrete matrix metalloproteinases that degrade the basement membrane and facilitate endothelial invasion. Rather than treating pericyte coverage as a late histologic badge of maturity, future studies should view mural biology as a dynamic control system that influences sprouting, hemocompatibility, leakage, and regression. In diabetes and chronic inflammation, pericyte dysfunction contributes to capillary instability and vascular rarefaction. The next challenge is therefore state-specific control: which pericyte phenotypes promote repair, which favor fibrosis or regression, and how can scaffold ligands, growth-factor sequencing, or mechanics be used to guide detachment, migration, and reattachment at the right time? Mechanistic support for this framework is persuasive, but direct indication-relevant datasets remain limited [101,102,103,104,105].

#### 3.2.3. Microvascular Fragments and Stromal Vascular Fraction

**Figure 9:**
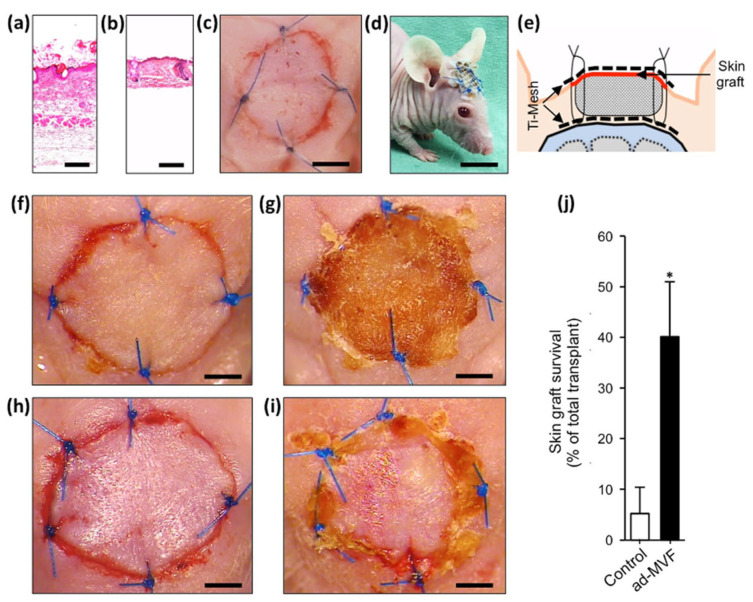
Early autologous split-thickness skin grafting on prevascularized versus non-seeded dermal matrices, including graft transfer, stereomicroscopy, and graft-survival quantification. (**a**–**e**) HE-stained sections of a skin graft before (**a**) and after (**b**) defatting. The autologous grafts are transferred onto implanted Integra and are secured with sutures, a sterile plastic dressing (**c**) and a titanized mesh (**d**,**e**). (**f**–**i**) Stereomicroscopy of non-seeded (**f**,**g**) and prevascularized (**h**,**i**) implants immediately (**f**,**h**) and 5 days (**g**,**i**) after skin grafting. (**j**) Quantification of skin graft survival (% of total transplant) 5 days after transplantation. Mean ± SEM, n = 8, * *p* < 0.05 vs. non-seeded control. Scale bars: (**c**) = 2.5 mm, (**d**–**g**) = 1.8 mm, (**h**–**i**) = 2 mm. Reprinted with permission from Ref. [74], 2018, Frueh et al.

As discussed, MVFs rapidly assemble into networks and improve perfusion [72,73,74,75,76]. Stromal vascular fraction (SVF) contains MSCs, ECs, fibroblasts, and macrophages. It has been used to promote angiogenesis, but it lacks the preassembled lumenized segments that give MVFs their ready-to-connect advantage [125,126,127,128]. Comparative studies show that MVFs engraft and restore blood flow better than SVFs and MSCs [75,76]. This comparison emphasizes a broader principle of tissue preservation in regenerative medicine and highlights the importance of cell quality within the tissue at the site of application. Clinically, adipose (fat) tissue is appealing because it is both readily available and abundant. Still, the extent to which each of the four sources of adipose tissue (i.e., MVF, SVF, and stromal) can be used successfully from a therapeutic standpoint remains unknown. Although there are some commonalities with MVFs, SVFs and stromal products that are currently being developed, future work should focus on evaluating them against the same criteria as those used in evaluating MVF, SVF and stromal products against their respective clinical endpoints (i.e., speed of connection, degree of success of the perfusion network, durability, ability to manufacture, flow stability, and immunogenicity) rather than simply comparing their results by means of non-standardized types of readout for evaluating their angiogenic activity. Although cell-based therapies using MVFs may currently be the most logical choice based on preclinical evidence, additional research is needed to develop standardized methods for processing and harvesting MVFs, SVFs, and stromal products [114,115,116,117,125,126,127,128]. Figure 9 shows the translational implication of MVF-based prevascularization: a dermal matrix that becomes vascularized earlier can support split-thickness skin grafting earlier than a non-seeded control in the same wound model [74].

#### 3.2.4. Beyond Blood Perfusion: Lymphatic, Immune, and Neural Coordination

Although this review focuses on functional blood perfusion, successful skin regeneration cannot be reduced to blood vessel formation alone. Native skin repair depends on coordinated interactions among vascular, lymphatic, immune, neural, stromal, and epidermal systems. Blood vessels provide oxygen, nutrients, and systemic access, but lymphatic vessels regulate interstitial fluid clearance, edema resolution, antigen transport, and immune-cell trafficking; immune cells determine whether the wound environment supports repair or persistent inflammation; and cutaneous nerves influence vascular tone, inflammation, keratinocyte behavior, fibroblast activity, sweat gland function, and sensory recovery [97,98,99,100,101,102,103,104,105,113,129,130,131,132]. Therefore, the aim of perfused skin engineering should not be only to create a blood-filled microvascular bed, but to generate a tissue environment in which vascular perfusion is coupled to fluid drainage, immune resolution, epidermal maturation, and sensory-functional recovery.

Lymphatic integration is particularly important because blood perfusion without drainage may worsen edema, increase diffusion distance, and destabilize graft integration. Newly formed blood vessels can deliver plasma and inflammatory cells into the construct. Still, without a parallel route for fluid clearance and immune-cell exit, the graft may become congested, swollen, and metabolically inefficient. Lymphatic vessels also provide an immune-surveillance route by transporting antigens and immune cells toward draining lymph nodes, and they participate in matrix remodeling during wound repair [129,130]. From an engineering perspective, this means that future vascularized skin substitutes should be evaluated not only for inflow and capillary perfusion, but also for interstitial fluid handling, lymphatic marker expression, edema control, and the presence of lymphatic-like drainage pathways. MVF-based strategies are particularly relevant in this regard because adipose-derived microvascular fragments may contribute not only to blood microvessel formation but also to the formation of lymphatic-like structures and to improved dermal matrix integration [73,74].

Immune regulation is equally central to the transition from early vascularization to durable regeneration. Acute inflammation is necessary for debris clearance, antimicrobial defense, and initiation of angiogenesis. Still, unresolved inflammation can produce a hostile environment characterized by excessive protease activity, oxidative stress, endothelial dysfunction, leaky vessels, fibrosis, and impaired re-epithelialization [97,98,120,124]. Macrophages should therefore be viewed not simply as inflammatory contaminants, but as dynamic regulators of the vascular niche. Early macrophage-associated signals can support endothelial activation and sprouting, whereas later pro-resolution programs contribute to matrix remodeling, pericyte recruitment, vessel stabilization, and barrier recovery [97,98,99,100,120,124]. This is especially relevant in diabetic, infected, ischemic, and irradiated wounds, where impaired immune resolution and vascular insufficiency reinforce each other. For engineered skin constructs, the translational question is therefore not whether inflammation can be eliminated, but whether the material, cellular composition, degradation products, and release cues can guide inflammation toward timely resolution and stable vascular remodeling.

Neural regulation adds another layer of complexity that is often underrepresented in vascularized skin models. Skin is a highly innervated organ, and sensory and autonomic nerves release neuropeptides and neurotrophic factors that influence vasodilation, inflammation, keratinocyte migration, fibroblast function, angiogenesis, sweating, pain, and barrier recovery [131]. Denervation, neuropathy, or impaired neurovascular signaling can delay healing and is particularly relevant in chronic wounds, including diabetic wounds, where vascular insufficiency and sensory dysfunction coexist [19,20,131]. In engineered skin, the absence of neural components may limit not only sensory recovery but also the ability to reproduce neuroimmune and neurovascular regulation. Although full innervation may not be a realistic early requirement for every implantable skin substitute, future models should at least define whether neural regulation is irrelevant, indirectly represented, or intentionally engineered into the platform. For skin-on-chip and preclinical qualification systems, incorporation of sensory neurons, Schwann cells, neuropeptide readouts, or neuroinflammatory stimulation may improve predictive value when pain, itch, inflammation, vasoregulation, or chronic wound biology is part of the intended use case.

These considerations suggest that the next generation of perfused skin constructs should be evaluated through an integrated “vascular–lymphatic–immune–neural” lens. In early-stage implantable grafts, the minimum requirement may remain rapid blood perfusion and graft survival; however, more advanced constructs should also address edema control, immune-cell trafficking, inflammatory resolution, neural compatibility, and restoration of skin-level functions. In perfused in vitro platforms, the same principle should guide model complexity: additional lymphatic, immune, or neural components should be included when they materially improve the biological question, disease relevance, or translational decision. The goal is not to make every model maximally complex, but to match biological completeness to the intended clinical or experimental purpose.

### 3.3. Advanced Biofabrication and Microfluidic Technologies

The field of advanced biofabrication is often described as a single area of development, but in reality, there are at least two distinct types of applications. The first type of application involves the fabrication of implantable skin substitutes that must withstand implantation and connect to the recipient’s native blood vessels. The second type of application is the construction of in vitro perfused platforms, such as the skin-on-chip (SoC) platform and similar systems [31,32,33,34,35,48,49,50], which provide a physical model of the skin for studying mechanistic processes leading to skin maturity or for using the model as a preclinical indication for skin substitute implants. While both types of applications utilize principles of vascular engineering, they differ in how they define the products being developed and in their success criteria. By maintaining this distinction, we can avoid mistakenly interpreting well-engineered in vitro systems as viable implants and better connect our available evidence to the products’ intended purpose. The primary extant evidence regarding SoC systems tends to center around their ability to measure and predict outcome, and the primary extant evidence regarding implanted skin substitutes emphasizes survival, connection to host circulation, and ease of use following surgery [5,11,12,13,14,15,16,17,26,27,28,29].

#### 3.3.1. Three-Dimensional Bioprinting and Sacrificial Approaches

**Figure 10:**
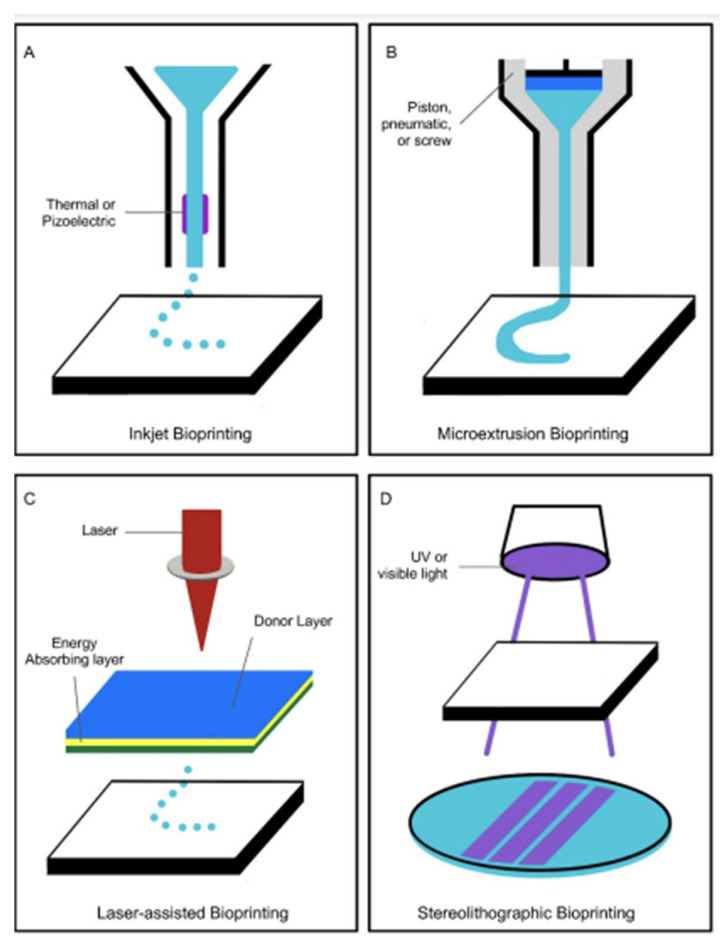
Four commonly discussed 3D bioprinting methods for vascularized constructs: inkjet, microextrusion, laser-assisted, and stereolithographic bioprinting. Bioprinting: inkjet (**A**), microextrusion (**B**), laser-assisted (**C**), and stereolithographic (**D**). Reprinted with permission from Ref. [51], 2023, Mir et al.

Bioprinting enables the precise placement of cell types and/or biomaterials in layers, allowing the development of skin analogs [37,38,39,94,95,96]. Bioinks (methacrylated hydrogels like GelMA) are crosslinked by photopolymerization, enabling cells to attach via RGD peptide sequences [90,91,110,111]; modifications (chemically) can tune their crosslinking kinetics, mechanics, and degradation. Sacrificing the bioink during printing (with material like alginate/agarose/polycaprolactone (PCL)/Pluronic F127) and subsequent removal leaves behind channels that are then vacated to furnish structural vessels; examples include filling/extruding GelMA with Pluronic F127, creating stable vascular networks, and using SilkMA/GelMA and methacrylated hyaluronic acid to manufacture microchannels (360 μm) that supported endothelial lining and fibroblast development; these constructs were tested for improved healing of wounds in vivo [39,40,41,94,95,96]. A critical trade-off exists between resolution and fidelity. Whereas both are important, it may be that speed, viable area, and acceptable behavior post-printing must also be considered. Current extrusion bioprinters will always be limited in creating true capillary channels (<100 μm); however, it is important to note that deposition precision does not guarantee biological fidelity once fibroblast contraction and/or matrix remodeling have begun. To that end, bioprinting should be assessed not only on geometric accuracy (Day 0) but also on its ability to facilitate flow distribution, control leakage characteristics, and accommodate reasonable inflow/outflow behaviors [42,43,44,45,46,47]. Figure 10 places the bioprinting discussion into an engineering map. Inkjet, microextrusion, laser-assisted, and stereolithographic approaches differ in how they deposit material, which also explains why resolution, cell stress, throughput, and channel geometry are difficult to optimize simultaneously [37,38,51].

**Figure 11:**
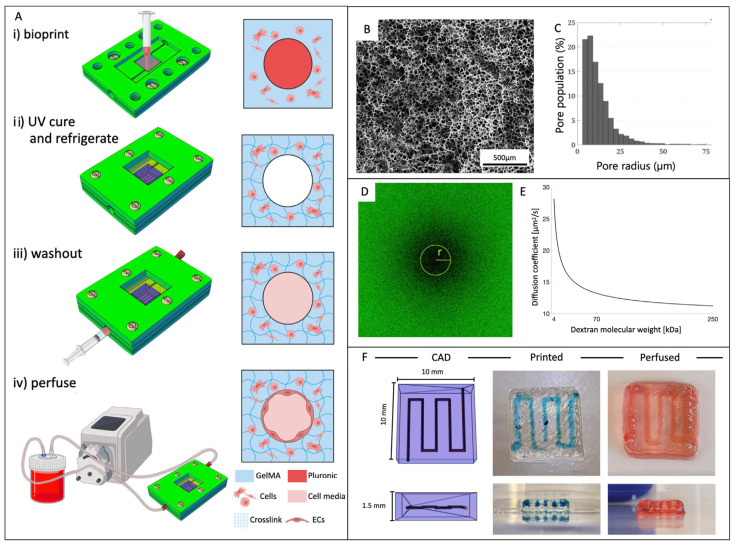
Multi-material and sacrificial bioprinting workflow for a perfusable vascularized 3D construct, including GelMA matrix formation, Pluronic sacrificial-channel removal, endothelialization, and perfusion. (**A**) Outlined protocol for fabrication of vascularized 3D constructs. (i) structure bioprinting inside the bioreactor; (ii) UV exposure to crosslink GelMA and refrigeration for dissolving the sacrificial material (PLU); (iii) washout of PLU by injecting cold fluid; (iv) connection to the perfusion circuit. (**B**) Representative SEM image of the internal microstructure of 8% GelMA and (**C**) pore size distribution. (**D**) Confocal image of a bleached sample during FRAP testing and (**E**) diffusion coefficient of 8% GelMA as a function of molecular weight of dextrans. (**F**) From left to right: CAD model of the vascular channel geometry, bioprinted structure (PLU in blue), and after dye perfusion. Reprinted with permission from Ref. [133], 2025, Maggiotto et al.

Laser-assisted and digital light processing (DLP)-assisted bioprinting has enabled the creation of structures at much higher resolutions than previous methods [37,38,39,40,41,42]. Methacrylated silk fibroin, printed using DLP, generated complete thickness skin constructs that were conducive to the growth of keratinocytes and fibroblasts; however, at present, no single bioprinting method adequately addresses all the combined needs: (1) creating structures with capillary-sized detailing (2) being capable of manufacturing areas of clinical importance (3) processing cells in a manner that is not destructive to them (4) providing hemocompatible interfaces and (5) manufacturing at the speed needed for use in a clinical setting. Accordingly, the emergence of hybrid bioprinting pipelines is becoming increasingly appealing [42,43,44,45,46,47]: printing large-volume tissues to provide area coverage, patterning small features with light, using sacrificial templating techniques to generate conduits, and ultimately subjecting these tissues to dynamic flow to produce the desired biophysical profile. New strategies/systems such as 2-photon polymerization and volumetric bioprinting show promise for addressing certain aspects of this problem; however, an important question is how to effectively combine the various bioprinting modes into a single biologically coherent workflow that is scalable to a clinical environment. Current evidence supports improved architectural control and wound healing in rodent models. Still, it does not yet provide sufficient evidence to demonstrate the durability of capillary-scale flow dynamics in tissue-engineered constructs manufactured at human-relevant scales [37,38,39,40,41,42,43,44,45,46,47,100,101,102,103,104,105,106,107,108,109]. Figure 11 shows a transferable sacrificial-channel workflow: a removable channel template is printed, the matrix is cured, the sacrificial phase is washed out, and the resulting channel is perfused. This is directly relevant to the manuscript’s discussion of convective transport and macro-to-micro access routes [133].

#### 3.3.2. Microfluidic and Skin-on-Chip Platforms

**Figure 12:**
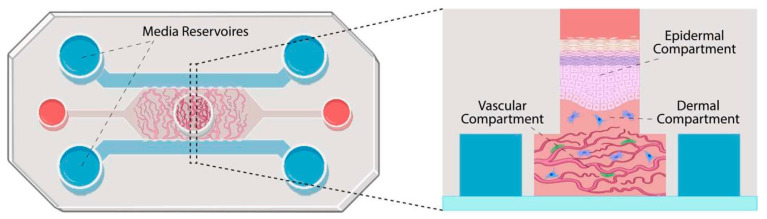
Schematic of an integrated microvascularized human skin-on-chip model, showing media reservoirs, vascular compartment, dermal compartment, and epidermal compartment. Reprinted with permission from Ref. [31], 2022, Jones et al.

Microfluidic devices enable precise control of fluid flow, shear stress, nutrient gradients, and cell distribution [31,32,33,34,35,57,59,62,63]. Therefore, skin-on-chip systems represent a valuable resource for studying various phenomena related to barriers, endothelium behavior, immune trafficking, disease transmission, drug delivery, etc., which take place in a dynamic environment by way of continuous perfusion and real-time monitoring as a result of the ability to integrate dermal and epidermal layers and vascular compartments. Continuous perfusion and real-time monitoring are achieved in these systems by incorporating dermal and epidermal layers with vascular systems [48,49,50]. Dual-compartment media allow keratinocytes to differentiate at an air–liquid interface and support the basal supply of endothelial growth factors; dynamic perfusion capability maintains tissue viability and barrier function. Despite this, a skin-on-chip system is not an implantable graft. The value of these systems lies in their controllability, observability, and reproducibility. Therefore, depending on the specific application, the question of application is relevant: does the device serve to model disease, evaluate therapy, mature a construct before implantation, or qualify a product for translation into a clinical environment? When all of these purposes are treated as interchangeable, it dilutes the engineering logic and the translational rationale for development. Innovative advances in microfabrication via soft lithography, PDMS micromachining, stereolithography, and injection molding allow for the scalable, precise production of chips with defined microchannel architectures. An example of innovative micro-precision 3D printing is a micro-precision 3D-printed chip that contains perfusable, capillary-like channels made from biocompatible resins, thereby avoiding PDMS absorption [32,33], while enabling the creation of six perfused chips in a high-throughput format. In these cases, perfusion increased nutrient supply to the chips compared with static culture, thereby maintaining improved barrier function and chip viability. Another type of design, which incorporates human keratinocytes, fibroblasts, and endothelial cells, was fabricated on a microfluidic chip; vascularization enhanced keratinocytes’ ability to undergo stratification and, therefore, to express differentiation markers. Present designs can vary significantly in material selection, flow characteristics, channel geometry, medium formulation, and functional outcomes. Hence, as skin-on-chip designs transition from development to regulatory conformity, uniform standards for assessing preclinical validity must be established [48,49,50,114,115,116,117]. There are minimum standards that must be established for barrier integrity, vascular patency, thrombogenicity, epidermal differentiation, inflammatory responsiveness, and repeatability at different laboratories if these devices are to provide a viable basis for preclinical qualification and therefore not simply a product-specific demonstration. Figure 12 clarifies the architecture of a skin-on-chip model as a non-implantable but informative platform. The device separates vascular, dermal, and epidermal compartments while enabling medium flow and controlled observation [31].

Microfluidics can also be used to create perfusable microvasculature structures in a hydrogel scaffold using endothelial cells via traditional vasculogenesis or hybrid methodologies. Each of these builds has been designed to mimic the circular cross-section of the vessel, have an apical–basal orientation of the cells within the channel, and provide continual perfusion for the transport of nutrients and waste products while simulating disease models [48,49,50,57,59,62,63,77,78]. When performing research with these device types, one can use several methodologies for creating the substrate supports, including microneedle-based removable molds, planar micromolding, and dissolvable sacrificial micromolding, with varying levels of control over the resulting channel geometry. Because of the controlled nature of the endothelial cell lining for these studies, they are frequently preferred for transport studies [57,59,62,63,78]. However, endothelial cell-lined channels do not fully replicate angiogenic self-assembly. Although vasculogenic and hybrid models produce networks with greater physiological relevance, they are less amenable to control over topology and interconnectivity. In addition, the question yet to be answered is not only how vascular morphology can be reproduced, but also how much vascular realism is appropriate for the investigation. A hybrid system consisting of self-assembled microvessels with patterned conduits is likely to be modulated by skin properties, including the presence of superior plexuses, deeper vessels, vertical connections, thrombogenicity, and possibly lymphatics. Ultimately, perfusion devices should be evaluated by how well they facilitate investigation and prediction, rather than solely by how well their geometry resembles the textbook representation of microvascular trees. The strongest scientific basis supports the use of these devices for mechanistic investigations and comparative evaluations, not for direct use in preclinical implantation [31,48,49,50].

#### 3.3.3. Prevascularization and On-Chip Vascular Maturation

**Figure 13:**
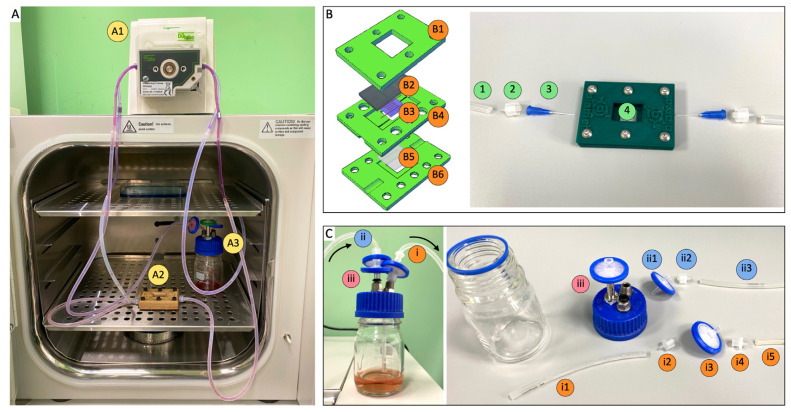
A custom perfusion system and a construct holder are used to maintain perfusable 3D constructs under controlled flow conditions. (**A**) Perfusion circuit composed of (A1) peristaltic pump, (A2) bioreactor, and (A3) cell medium reservoir. (**B**) Left: bioreactor design. (B1) top blocking frame, (B2) PDMS layer, (B3) vascularized construct, (B4) bioreactor body, (B5) coverslip with PDMS coating, (B6) bottom supporting plate. Right: connection to perfusion circuit. (1) silicone hose, (2) male luer lock, (3) plastic flexible tube tip 22G, (4) vascularized construct within bioreactor. (**C**) Reservoir configuration. (i) suction section: i1. Silicone hose, i2. Male luer lock, i3. 0.22 µm filter, i4. Female luer lock, i5. Silicone hose. (ii) pouring section: ii1. 0.22 µm filter, ii2. Male luer lock, ii3. Silicone hose. (iii) 0.22 µm air filter. The inner diameter of all silicone hoses was 0.51 mm. Reprinted with permission from Ref. [133], 2025, Maggiotto et al.

Two- to four-week cultures of prevascularized synthetic skin structures are produced to provide vascularized synthetic skin upon implantation. The approach combines Huvecs and pericytes with fibroblasts, using a collagen or fibrin scaffold to produce a capillary-like network that will quickly interweave with the hematopoietic cell layer of host vessels [30,36,56]. The skin chip technology facilitates perfusion during prevascularization of endothelial cells. It encourages them to mature to a higher degree of barrier function in the presence of perfusion and flowing fluid. The utility of the perfused microvasculature for modeling diseases has been established through experimentation with atopic dermatitis and psoriasis using perfused microvascularized skin chips. Exposure of the skin chips to anti-inflammatory cytokines alters epidermal cell morphology and changes the expression of cellular markers indicative of inflammatory responses. Flow conditioning of the dermis will tighten the junctions between adjacent endothelial cells, identify regions of weakness in blood vessels before surgery, and promote greater organization of the dermal matrix. However, maturation of dermal endothelial cells in an environment with flow will create challenges in terms of translational outcome [50,114,115,116,117,133] such as contamination risks, costs associated with manufacture, bioreactor duplication ability, and development of the construct, as well as determining the acceptable time between the manufacture and implantation of the construct. On-chip maturation may be especially justifiable in reconstructive settings that tolerate longer preparation, whereas large acute burns may favor simpler products that can be deployed quickly. The field now needs comparative studies to determine which properties must be achieved ex vivo and which are more efficiently entrusted to the host. On-chip maturation is therefore best understood not as a universal requirement, but as a tunable manufacturing step whose value depends on indication, product format, and logistics. It can stress-test barrier function and flow tolerance before surgery, but it does not, by itself, solve recipient macrovascular coupling. Taken together, current biofabrication and microfluidic approaches differ not only in how precisely they create vascular geometry, but also in whether they are presently stronger as implant-enabling technologies or as model- and qualification-oriented platforms (Table 7). Figure 13 illustrates the practical manufacturing burden of ex vivo flow conditioning. The perfusion circuit, tubing, construct holder, reservoir, and pump are all part of the maturation and quality-control environment, not merely background equipment [133].

## 4. The Translation Gap: From Bench to Bedside

Despite encouraging preclinical results, engineered skin substitutes rarely achieve the rapid, stable vascularization necessary for clinical success. Part of the translation gap arises because laboratory success and surgical success are defined differently. Histologic vessel density, marker expression, or short-term survival may be scientifically encouraging. Yet, clinicians need stable wound closure, resistance to infection and contraction, practical handling, predictable timing, and performance in compromised hosts [18,19,20,21,129,130,134,135,136,137]. Translation will remain slow until the field adopts a minimum translational dataset for vascularized skin constructs [50,114,115,116,117]. At a minimum, that dataset should include time to active perfusion, stability of patency, construct-level hemocompatibility or thrombogenicity, workable inflow–outflow balance, barrier recovery, infection resilience, handling and fixation characteristics, durability during follow-up, and a potency framework that links release assays to clinical intent. Constructs should be compared not only with one another, but also against the standard of care they aim to displace or augment. Across most strategy classes, the available evidence is still dominated by short-duration preclinical studies, especially in rodents [134,135,136,137]. The minimum translational dataset proposed for vascularized skin constructs is summarized in Table 8.

### Why Promising Perfusion Strategies Fail During Clinical Translation

The limited clinical translation of perfused or prevascularized skin constructs does not usually result from a single missing technology. Rather, it reflects a mismatch between the encouragement of experimental readouts and the combined biological, surgical, manufacturing, and regulatory demands of clinical use. Many strategies can increase endothelial marker expression, vessel-like morphology, or short-term vascular density in vitro or in small-animal models. Still, these outcomes do not necessarily predict whether a construct will survive implantation, integrate with a compromised wound bed, tolerate blood entry, maintain distributed flow, resist infection, and provide durable skin-level function. For this reason, translational failure should be understood as a multi-domain problem rather than as a simple failure of vascularization [11,12,13,14,15,16,17,18,19,20,21,26,27,28,29,49,50,57,58,59,60,61,62,63,77,78,129,130,131,132,134,135,136,137,138,139].

First, immune response is a major determinant of whether a vascularized construct becomes integrated, fibrotic, infected, or rejected. Biomaterials, decellularized matrices, degradation products, allogeneic cells, residual processing agents, and microbial contamination can all trigger host responses that alter endothelial survival and vascular remodeling. A limited early inflammatory response may support angiogenesis, but persistent inflammation can destabilize nascent vessels, increase permeability, promote edema, and impair epidermal barrier recovery. This is especially important in diabetic, ischemic, irradiated, infected, or chronic wounds, where macrophage behavior, endothelial function, and stromal remodeling are already abnormal [18,19,20,21,97,98,99,100,101,102,103,104,105]. Thus, immune compatibility should not be evaluated solely by the absence of acute cytotoxicity; it should be assessed alongside vascular stability, macrophage transition, edema control, and durable graft integration.

Second, graft integration remains more difficult than vascular formation alone. A construct may contain preformed lumens or endothelial networks, yet still fail if it does not establish workable inflow and outflow, if the interface with the wound bed is poorly aligned, or if blood flow is shunted through a few low-resistance channels. Integration also requires mechanical conformity, fixation, resistance to shear during dressing changes, dermal–epidermal coupling, lymphatic or fluid drainage, and compatibility with the local microbial and inflammatory environment. Therefore, vascularized skin substitutes should not be judged only by whether vessels are present inside the construct, but by whether those vessels support stable tissue incorporation and downstream wound repair [36,56,129,130,131,132].

Third, scalability changes the product’s biology and engineering. A construct that is viable at the millimeter or subcentimeter scale may fail when scaled up to clinically relevant wound areas because diffusion distances, oxygen demand, channel resistance, pressure gradients, fluid drainage, and handling requirements all change simultaneously. Increasing construct size also increases the need for reproducible vascular hierarchy, venous outflow, robust mechanical integrity, and standardized surgical deployment. In this sense, scale-up is not simply the production of a larger version of the same prototype; it is a different design problem involving transport, biomechanics, sterility, process control, packaging, storage, and operating-room workflow [5,37,38,39,40,41,42,43,44,45,46,47,48,49,50,51,60,61,79,80,81,82,100,101,102,103,104,105,106,107,108,109,133].

Fourth, manufacturing and regulatory constraints often emerge late, after promising preclinical data have already been generated. Living or vascularized skin constructs may combine cells, scaffolds, bioactive molecules, printed architectures, perfusion conditioning, and patient-specific handling, making them difficult to classify, release-test, sterilize, store, transport, and reproduce. Cell-based products require validated cell sourcing, donor screening, expansion protocols, potency assays, batch-to-batch comparability, and defined release criteria. Biomaterial-containing products also require biocompatibility, degradation, residuals, mechanical performance, and safety evaluation within a risk-management framework. Without early definition of the intended mechanism of action and a corresponding potency or performance assay, it becomes difficult to connect manufacturing quality to clinical efficacy [50,114,115,116,117].

Finally, many approaches fail to translate because the preclinical evidence package is not aligned with the intended clinical indication. Acute burns require rapid availability, infection control, handling strength, and early graft take. Chronic diabetic or ischemic wounds require performance in a poorly vascularized and inflamed host bed. Reconstructive defects may tolerate longer manufacturing timelines but require contour, thickness, and durable integration. Therefore, the same vascularization strategy cannot be considered universally translatable unless it is tested under indication-relevant conditions. Future studies should define the target clinical scenario early and then align the construct design, animal model, perfusion readouts, immune assessment, manufacturing strategy, and regulatory pathway with that scenario [18,19,20,21,49,50,113,114,115,116,117,129,130,131,132,134,135,136,137,138,139].

A clinically useful translational pathway also cannot be indication-agnostic. Acute burns prioritize immediate coverage, infection control, and rapid graft take; diabetic and ischemic wounds demand performance in a host bed already compromised by endothelial dysfunction, inflammation, neuropathy, and poor oxygen delivery [18,19,20,21,113]; irradiated tissue adds stromal damage and impaired remodeling; reconstructive defects may tolerate greater manufacturing complexity if contour, thickness, and durable integration are improved. A construct that is promising for one scenario may therefore be poorly matched to another, so benchmarking should increasingly be indication-specific rather than universally generic [114,115,116,117,129,130,134,135,136,137]. The same logic should guide strategy choice throughout the field: a platform optimized for fast deployment is not automatically the best platform for chronic wound biology, and a construct engineered for maximal biologic completeness may be unnecessarily complex for routine acute coverage.

First, species differences between animal models and humans are profound. Mice and rats have thin skin and rapid healing, and their immune systems differ markedly from those of humans [134,135,136,137]. Microvascularized skin substitutes that perform well in rodents may fail in pigs or humans due to differences in skin thickness, immune response, and mechanical stress [49]. Many studies on immunocompromised animals omit critical immune–vascular interactions. Preclinical models also rarely reproduce comorbidities such as diabetes, peripheral vascular disease, or aging that blunt angiogenesis in patients. Rather than searching for a single perfect model, the field likely needs a tiered evidence strategy: mechanistic work in controllable in vitro systems; immunocompetent small-animal studies for early biologic screening; large-animal or surgically relevant models for thickness, biomechanics, and handling; and ex vivo human or humanized systems for patient-relevant validation. Longitudinal perfusion imaging should complement endpoint histology to distinguish early connection, later regression, and delayed remodeling [58,59,60,61].

**Figure 14:**
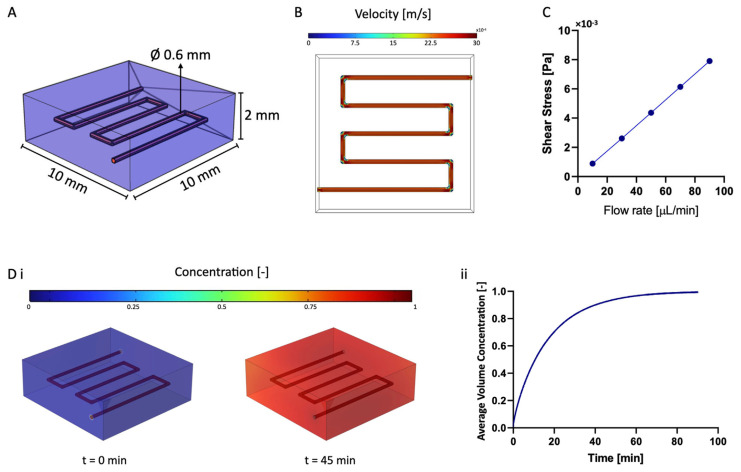
Computational analysis of a perfusable 3D channel network, including channel dimensions, velocity distribution, shear stress as a function of flow rate, and solute diffusion over time. (**A**) 3D model used for numerical simulations: the vascular channel was the free domain, while GelMA was the porous domain. (**B**) Flow pattern within the vascular channel. (**C**) Result of the parametric sweep to estimate the value of wall shear stress by varying the flow rate. (**D**) Diffusion of diluted species from the vascular channel to the surrounding GelMA: the target concentration was reached after approximately 45 min and was maintained over time, as shown in (ii). Reprinted with permission from Ref. [133], 2025, Maggiotto et al.

Second, the scale and geometry of engineered constructs matter. Laboratory constructs often measure only a few centimeters square; full-scale human grafts require orders of magnitude greater vascular surface area. Achieving uniform perfusion across large areas is challenging, and perfusion circuits may create shear-stress gradients that affect differentiation [60,61,79,80]. Geometry also determines how a graft is handled in the operating room. Thick, highly hydrated constructs may be biologically sophisticated yet difficult to trim, secure, or contour to irregular wound beds. In contrast, very thin constructs may integrate more readily but fail to provide sufficient dermal bulk or mechanical resilience. Scaling up also exposes a macro-to-micro integration problem. A construct may contain a promising microvascular network yet lack a practical route for host inflow and outflow to reach it quickly enough [5,37,38,39,40,41,42,43,44,45,46,47,100,101,102,103,104,105,106,107,108,109]. Modular perfused units, open-channel designs, and surgically assisted bridging strategies may help connect engineered microvessels to recipient macrovasculature, but these concepts remain immature. Scale-up is therefore not merely making the same construct larger; it alters transport, mechanics, manufacturing logistics, and surgical workflow simultaneously [50,114,115,116,117]. Large burns may emphasize area, deployment speed, and simple fixation, whereas focal reconstructive defects may justify more elaborate vascular integration if contour and thickness can be improved. Figure 14 connects scale with flow design. Numerical simulation of channel velocity, shear stress, and diffusion shows why increasing construct size or changing channel geometry is not just a fabrication issue but also a transport and hemocompatibility problem [60,61,79,80,133].

Third, immune–vascular integration is inadequately modeled. Most in vitro systems lack immune cells, and even those that incorporate macrophages or dendritic cells cannot capture the dynamic immune shifts seen in vivo [97,98,99,100,134,135,136,137]. Skin-on-chip models integrating vascular, immune, and nerve components show promise; they incorporate Langerhans cells, dendritic cells, and macrophages to emulate immune responses, and co-culture strategies improve barrier properties and cytokine signaling. Yet immune–vascular crosstalk remains simplified, and standardized activation protocols are lacking [48,49,50,97,98,99,100]. The absence of lymphatic and neural components further limits predictive power. Lymphatics influence edema control and immune-cell trafficking [129,130], while innervation affects vascular tone, sweat gland regulation, and aspects of wound healing [131]. Likewise, the microbiologic dimension of skin is often simplified even though infection and dysbiosis are central to chronic wound failure [113,132]. The goal is not maximal complexity for its own sake, but strategic completeness: adding the missing components that materially improve prediction for the question being asked.

Fourth, regulatory and manufacturing challenges limit translation. Many vascularization strategies rely on complex, custom-made devices or materials that are difficult to scale and sterilize. Variability in decellularized ECM, cell sources, and bioprinting processes affects reproducibility. Regulatory agencies require robust data on safety, batch consistency, and long-term outcomes [138]. Manufacturing discipline will therefore be just as important as biologic innovation. Living skin constructs require validated cell sourcing, reproducible scaffold fabrication, sterile assembly, in-process monitoring, and clear release criteria before clinical use [139]. Translation will depend on building manufacturing strategies early, not after efficacy has been demonstrated. Potency assays, comparability rules after process changes, storage conditions, cost of goods, and staffing requirements all influence whether a vascularized construct can leave the academic prototype stage [50,114,115,116,117]. Partnerships between engineers, biologists, clinicians, and industry will be necessary to move perfused skin constructs toward clinical trials. At present, this remains one of the largest gaps between conceptual promise and publishable translational confidence.

## 5. Roadmap: Top Five Grand Challenges for the Next Decade

Based on the analysis above, five priorities emerge that are better understood as a connected research architecture than as isolated technical hurdles. Each priority is framed as an unresolved question because the field now needs experimentally tractable problems rather than broad aspirations. For each, the critical issues are not only scientific interest but also which evidence level is missing, which models are most suitable, which readouts should define progress, and which translational bottleneck would actually be solved. The roadmap below is, therefore, intentionally benchmark-oriented [5,26,27,28,29,50,114,115,116,117].

What should count as healthy perfusion? The field needs shared definitions that go beyond vessel density or occasional blood filling [58,59,60,61]. The most informative benchmark set will combine controlled flow systems with in vivo validation. It should include lumen continuity, red blood cell or tracer transit, oxygen rescue across the construct thickness, leakage or permeability, thrombus burden, distribution of flow across the network, inflow–outflow balance, and persistence of patency during remodeling. The best first test beds are paired systems: perfused in vitro constructs for mechanistic measurement, followed by indication-relevant in vivo confirmation of host connection and durability. The translational payoff is decisive—comparable go/no-go criteria that distinguish visually vascularized constructs from products genuinely approaching clinical usefulness [50,114,115,116,117].

How can scaffold dynamics be synchronized with vascular development? Materials must open, soften, degrade, and signal in step with cellular invasion and vascular maturation rather than on a fixed clock [83,120,121,124]. The most informative studies will couple responsive matrices to time-resolved measurements of porosity change, invasion depth, flow initiation, oxygen rescue, contraction, and matrix replacement. Mechanistically, this question is best addressed through integrated material–biology platforms that combine imaging, transport modeling, and staged implantation studies. Solving it would create constructs that survive the early ischemic interval without sacrificing later mechanical integrity or architectural control. Acute-coverage products and chronic wound products may require different timing logic, so benchmark sets should reflect indication as well as material chemistry.

How should immune, mural, lymphatic, and vascular cues be orchestrated? Productive perfusion requires more than endothelial sprouting; it depends on macrophage timing, pericyte state, edema control, and matrix turnover [97,98,99,100,101,102,103,104,105,131,132]. Hypothesis-driven models should therefore track macrophage transitions, pericyte recruitment, lymphatic analogs, and barrier maturation in parallel rather than as separate subprojects. The best platforms for this challenge will combine reductionist in vitro assays with immunocompetent, disease-relevant in vivo models to distinguish early inflammatory benefit from later vascular instability. Progress here would directly address one of translation’s hardest problems: why constructs that perform well in healthy hosts often underperform in chronic, ischemic, or infected wounds [18,19,20,21,97,98,99,100,101,102,103,104,105,113,131,132,138,139].

How can hierarchical vascular networks be fabricated and connected at a clinically relevant scale? Printing capillary-scale features, maintaining viable areas, providing reliable venous outflow, and bridging engineered microvessels to host inflow and outflow are linked problems [37,38,39,40,41,42,43,44,45,46,47,100,101,102,103,104,105,106,107,108,109]. Hybrid fabrication, sacrificial templating, flow conditioning, and surgically assisted integration strategies should be compared on common metrics of scale, patency, handling, anastomotic feasibility, thrombosis resistance, and resistance to shunting or collapse. The right test sequence will likely move from controlled perfusion platforms to surgically relevant large-animal models [49,50]. This is the challenge that will determine whether the field moves beyond elegant microdevices toward grafts capable of closing large wounds, while still enabling more elaborate solutions for smaller reconstructive defects.

Which human platforms are predictive enough to qualify products before implantation? Skin-on-chip, bioprinted constructs, organoids, and ex vivo human systems should be developed as standardized test beds rather than demonstrations of complexity [31,32,33,34,35,37,38,39,40,41,42,43,44,45,46,47,48,49,50,51,133]. Validation requires harmonized cell sourcing, media and flow protocols, vascular and barrier readouts, and benchmarking against clinical biopsies, graft behavior, and indication-specific outcomes. Their greatest value will come from serving as decision platforms: identifying which constructs merit scale-up, which fail because of transport or immune liabilities, and which design rules transfer across products. The payoff would be a more rational translational pipeline in which implantable products are filtered, optimized, and de-risked in human-relevant systems before costly animal or clinical studies [50,114,115,116,134].

## 6. Conclusions and Future Outlook

The central claim of this review is that perfusion—not vessel density alone—is the master constraint in skin tissue engineering. Constructs must first survive the ischemic interval, then rapidly connect, tolerate blood entry, and remodel into stable, skin-specific microvasculature with functional inflow–outflow behavior. This sequence clarifies why so many individually promising strategies remain insufficient when used in isolation. Bioactive materials, cell therapies, MVFs, bioprinting, and microfluidic maturation each solve different parts of the problem. Still, there is no substitute for an integrated design logic that links oxygen rescue, host connection, hemocompatibility, immune timing, and manufacturability.

A second major conclusion is that implantable skin substitutes and perfused in vitro platforms should be linked but not conflated. Skin-on-chip systems are not the product itself; they are powerful tools for mechanism discovery, maturation studies, and preclinical qualification. Third, translation will depend on shared functional benchmarks, explicit evidence hierarchies, and indication-specific development pathways. The field should increasingly judge progress by time to perfusion, flow stability, barrier recovery, resistance to infection and contraction, and durable wound closure in clinically relevant settings. As a critical narrative review, the aim here has been not to duplicate recent platform catalogs but to reorganize them around the stricter question of which interventions truly shorten the path to durable, clinically useful perfusion. If these criteria guide study design, the next decade can move cutaneous tissue engineering away from descriptive vascularization and toward perfused, clinically deployable skin constructs.

## Figures and Tables

**Table 1:** Implantable skin substitutes versus perfused in vitro platforms: distinct application spaces, success criteria, and translational endpoints [11,26,31,32].

Platform Class	Primary Purpose	Requires Implantation?	Primary Source of Perfusion/Transport	Core Success Criteria	Typical Use Setting	Main Translational Bottleneck	Why It Matters in This Review
Conventional implantable skin substitutes (acellular or minimally cellular)	Rapid wound coverage; dermal replacement; wound-bed preparation; support for host-driven repair	Yes	Initial diffusion from the wound bed, followed by host angiogenesis/neovascularization	Early graft take, host vascular ingrowth, infection resistance, handling/fixation, barrier restoration	Acute burns; staged reconstruction; temporary or permanent dermal replacement	Slow vascular ingrowth in thicker constructs; limited biologic control in compromised wound beds	Provides the clinical baseline comparator against which more complex vascularization strategies should be judged
Prevascularized implantable skin substitutes	Shorten the ischemic interval by providing a ready-to-connect microvascular bed before implantation	Yes	Preformed lumens or vascular networks that must inosculate with host circulation	Reduced time to functional perfusion; survival of embedded cells; larger perfused tissue fraction; improved integration and wound closure	Advanced full-thickness wound repair; chronic defects; translational preclinical graft studies	Scale-up, storage, release criteria, donor/cell-source variability, thrombosis, and flow tolerance after blood entry	Represents the closest direct test of whether engineered vasculature improves implant performance in vivo
Bioprinted implant-ready skin constructs	Spatially organize cells, matrices, and conduits in a patient- or defect-specific graft format.	Yes	Printed channels, self-assembled microvessels, or hybrid channel-plus-network designs that must couple to host flow	Balance of area coverage, structural fidelity, perfusion potential, surgical usability, and post-implant remodeling	Reconstructive defects; customized graft fabrication; preclinical implant development	Trade-off among resolution, build speed, viable area, capillary realism, and macro-to-micro integration	Highlights that geometric precision alone is not enough unless it leads to clinically usable perfusion
Vascularized in vitro skin models (non-chip formats, including bioreactor-matured constructs)	Mechanistic study of vascularized skin biology; construct maturation; controlled hypothesis testing	No (unless later used as a maturation step before implantation)	Medium exchange, imposed perfusion, or controlled culture transport rather than native blood flow	Stable viability; reproducible vascular organization; interpretable biology; controllable experimental conditions	Mechanistic skin biology; maturation studies; comparative testing of materials and cells	Limited physiologic completeness; protocol variability; uncertain transferability to implant performance	Serves as an intermediate platform for understanding vascular behavior without being mistaken for a final clinical product
Skin-on-chip/perfused skin microphysiological systems (MPS)	Disease modeling; drug and cosmetics testing; barrier/transport studies; mechanistic discovery under controlled flow	No	Externally imposed microfluidic perfusion with defined flow rates and channel architecture	Reproducible barrier and vascular function; assay robustness; controllability; disease relevance; readout sensitivity	Nonclinical testing; toxicology; pharmacology; human-relevant mechanistic studies	Standardization, throughput, material–device effects, regulatory qualification, and inter-laboratory reproducibility	Clarifies that these systems are discovery and qualification tools, not direct substitutes for implant efficacy data
Preclinical qualification platforms (human-relevant decision platforms)	De-risk candidate products before animal studies or clinical translation; create go/no-go decisions	No	Controlled perfusion, ex vivo transport, or platform-specific dynamic testing	Predictive power; comparability across candidates; linkage between platform readouts and intended clinical use	Down-selection of lead constructs; potency framework development; translational benchmarking	Validation against clinically relevant outcomes; harmonized benchmarks; evidence transferability	Supports the manuscript’s argument that translation needs better qualification logic, not just more vascular-looking constructs

**Table 2:** Strategies that “buy time” before stable perfusion in engineered skin [5,56,58,60].

Time-Buying Strategy	How Does It Buy Time Before Stable Perfusion	What Protects First	Temporal Contribution	Main Liabilities	Best-Fit Use Context
Open transport channels/perfusable conduits	Creates low-resistance paths for medium, exudate, oxygenated fluid, or early blood/tracer entry into the construct, reducing reliance on pure diffusion across the full thickness	Central cell viability and transport to the construct core	Immediate transport advantage; can reduce the earliest diffusion bottleneck even before full capillary-level perfusion is established	Channels may remain non-endothelialized, promote shunting, weaken mechanics, or fail to connect meaningfully to host flow	Thicker dermal compartments; hybrid printed or templated constructs; systems intended for later vascular coupling
Lower initial metabolic load	Reduces oxygen consumption by lowering early cell density, staging cellularization, or delaying highly demanding functions until perfusion is established	Whole-construct survival window during the ischemic interval	Extends tolerance of the avascular phase during the first post-implant period	May weaken early matrix deposition, slow biologic maturation, or make the graft less skin-like at implantation	Acute-coverage constructs; staged products; early proof-of-concept systems
Temporary oxygen supplementation (oxygen-releasing or oxygen-carrying systems)	Locally supplies dissolved oxygen or generates oxygen while the host connection develops.	Metabolically vulnerable cells in poorly perfused regions, especially the construct core	Short bridging window for the early post-implant phase; duration is material-dependent	Finite oxygen payload, burst release, reactive by-products, pH control issues, and uncertain scale-up or in vivo reproducibility	Adjunct for acute ischemic management; best used together with channeling or prevascularization rather than as a stand-alone vascular solution
Pre-established lumens/prevascularized microvascular networks	Provides ready-to-connect vascular structures that can inosculate faster than de novo host sprouting	Distributed tissue regions that would otherwise wait for slow host vessel penetration	Can shift blood-entry timing from weeks toward days when host coupling is efficient	Manufacturing complexity, cell-source variability, storage constraints, thrombosis risk, and instability after first blood entry	Cell-rich full-thickness grafts; advanced dermal substitutes; reconstructive settings that tolerate higher manufacturing complexity
Ex vivo perfusion conditioning/pre-implant flow maturation	Exposes the network to controlled flow before implantation, improving endothelial readiness, barrier behavior, and detection of weak regions	Vascular integrity at the transition from static culture to real flow	Indirect time gain by reducing early post-anastomotic failure, leakage, or collapse	Adds manufacturing time, sterility burden, release-testing complexity, cost, and limited suitability for urgent deployment	Planned reconstruction; products with acceptable pre-implant maturation windows
Surgically assisted host coupling/direct inflow-shortening approaches.	Shortens the path between engineered microvasculature and recipient blood supply through vascular pedicles, loops, channel-guided coupling, or staged prefabrication	Large or thick constructs that cannot tolerate waiting for spontaneous host ingrowth	Potentially immediate or near-immediate macro-to-micro access to flow	Surgical complexity, indication selectivity, added morbidity, workflow burden, and limited routine applicability	Focal reconstructive defects; staged reconstruction; experimental large-format graft integration

**Table 3:** Operational benchmark set for functional perfusion in engineered skin [58,59,60,61].

Perfusion Criterion	What Should Be Measured	Minimum Acceptable Evidence	Stronger/Preferred Evidence	Suitable Assays or Readouts	Main Interpretation
1. Anatomical lumen readiness	Whether endothelial structures form continuous, open, lumenized pathways rather than isolated cords or blind-ended clusters.	Continuous endothelialized lumens across a meaningful construct depth.	3D lumen continuity with endothelial junctions, basement-membrane deposition, and mural-cell/pericyte support.	3D confocal imaging, optical sectioning, CD31/VE-cadherin staining, lumen reconstruction, pericyte markers, and basement-membrane markers.	Shows that the construct is structurally prepared for perfusion, but does not by itself prove flow.
2. Access to flow	Whether the engineered network can connect to host vessels or to an imposed perfusion circuit.	Demonstrable entry of medium, blood, tracer, or contrast into the engineered vascular space.	Stable connection with both inflow and outflow, without immediate collapse or disconnection.	Intravital microscopy, angiography, micro-CT angiography, circuit pressurization, flow-start verification, and vascular casting.	Separates vascular-looking constructs from constructs that are actually accessible to flow.
3. Blood flow or tracer transit	Whether red blood cells, fluorescent tracers, microspheres, or perfusate move through the network.	Movement of RBCs or tracer through at least part of the engineered network.	Quantified distributed transit through central and peripheral regions, with repeated measurements over time.	RBC tracking, fluorescent dextran perfusion, microsphere perfusion, live imaging, particle tracking, and perfusion mapping.	Directly supports a claim of perfusion rather than static vessel formation.
4. Flow distribution and avoidance of shunting	Whether the flow reaches broad regions of the construct rather than bypassing most of the tissue through a few large channels.	Evidence that flow is not limited to peripheral regions or a single dominant conduit.	Branch-level or region-level perfusion maps showing distributed transport across the intended tissue area and depth.	Perfusion heat maps, particle tracking, tracer distribution analysis, velocity mapping, and pressure-drop analysis.	Prevents false-positive interpretation of “perfusion” when only a small privileged subnetwork is perfused.
5. Oxygen delivery and metabolic rescue	Whether perfusion reduces hypoxia and sustains viable cells across clinically relevant construct thickness.	Reduced hypoxic core or improved viability compared with non-perfused controls.	Quantified oxygen gradients, reduced HIF-1α/hypoxia-marker signal, preserved ATP/metabolic activity, and improved viability through the full construct depth.	Oxygen probes, phosphorescence oxygen sensing, hypoxia dyes, HIF-1α staining, viability gradients, lactate/glucose consumption, metabolic assays.	This is the most direct test of whether perfusion is rescuing tissue rather than only filling vascular spaces.
6. Endothelial barrier and leakage control	Whether the vascular lining remains attached and limits excessive leakage during flow.	No catastrophic leakage or endothelial detachment after flow initiation.	Stable or improving permeability profile during maturation, with preserved junctional markers.	Dextran permeability assays, albumin leakage, VE-cadherin staining, live barrier imaging, and edema mapping.	Excess leakage reduces oxygen efficiency, promotes edema, and destabilizes graft integration.
7. Hemocompatibility and thrombosis resistance	Whether the network tolerates blood contact without rapid clotting or occlusion.	No immediate flow collapse, gross thrombus formation, or extensive platelet/fibrin accumulation.	Low thrombus burden under dynamic blood exposure and maintained patency after repeated or prolonged perfusion.	Whole-blood loop assays, ex vivo shunt models, platelet/fibrin staining, clot burden scoring, and occlusion-time measurement.	Functional perfusion fails if the network thromboses at the first blood entry.
8. Vessel stability and patency persistence	Whether perfused vessels remain open and functional during remodeling.	Patency persists beyond transient early filling.	Longitudinal evidence of stable perfusion, mural support, reduced regression, and maintained lumen structure over days to weeks.	Repeated tracer studies, longitudinal intravital imaging, histology–function correlation, mural-cell coverage, and basement-membrane staining.	Distinguishes temporary blood entry from durable functional perfusion.
9. Inflow–outflow balance and drainage	Whether the construct supports workable inflow and outflow without congestion, pooling, or edema.	No severe one-sided inflow, fluid trapping, or gross congestion.	Quantified outflow, pressure-drop behavior, reduced edema, and evidence of venous or lymphatic drainage support where relevant.	Outflow collection, pressure-drop analysis, edema scoring, venous-side imaging, lymphatic markers, tissue-fluid mapping.	Blood entry alone is insufficient; without exit and drainage, perfusion becomes unstable or nonfunctional.
10. Graft-level functional outcome	Whether vascular function translates into improved skin repair.	Improved viability, graft take, or wound closure compared with non-vascularized controls.	Durable graft survival, epidermal barrier recovery, reduced necrosis, reduced contraction, improved integration, and performance in indication-relevant models.	Graft survival/take, wound closure kinetics, histological integration, TEWL/TEER, epidermal differentiation markers, infection/chronic wound models.	Connects perfusion to clinically meaningful skin function rather than treating perfusion as an isolated engineering endpoint.

**Table 4:** Cross-category benchmarking of current vascularization strategies against staged perfusion endpoints and translational filters [26,37,83,97].

Strategy Class	Main Vascular Logic	Internal Network Formation	Host Connection/Inosculation	Blood-Entry/Flow Tolerance	Perfusion Stability/Fluid Handling	Manufacturability/Scale-Up	Best-Fit Application Space	Current Evidence Maturity
Scaffold architecture, topography, and porosity	Creates invasion space, transport paths, and cell-guiding geometry	Moderate—supports endothelial infiltration, lumen access, and depth of organization	Conditional—helps only if border interconnectivity and wound-bed access are adequate	Limited alone—rarely ensures hemocompatible flow without endothelialization or channels	Moderate to strong—strongly affects drainage, edema handling, and resistance to collapse	Generally favorable, but the architecture–mechanics coupling becomes harder at large areas and thickness	Foundational design variable for most implantable constructs	High preclinical maturity; low standardization for dynamic flow endpoints
Tuned degradation/responsive biomaterials	Opens space and changes mechanics in step with invasion and remodeling	Moderate—facilitates progressive invasion and matrix replacement	Indirect—may shorten the connection only if the scaffold opening occurs before ischemic damage accumulates	Conditional—poor timing can promote collapse, leakage, or loss of support	Strong potential—can preserve structure early and release constraints later	Moderate; requires reproducible chemistry, control of by-products, and timing logic	Implantable grafts needing staged remodeling, especially indication-specific designs	Moderate in vitro and rodent evidence; limited translational benchmarking
Protein-loaded bioactive scaffolds	Supplies pro-angiogenic cues to drive host sprouting or endothelial activation	Moderate—can accelerate sprouting and endothelial recruitment	Limited to moderate—depends heavily on host competence and local factor retention	Limited—often increases vessel number more than flow competence	Weak if used alone—may yield immature, hyperpermeable, or poorly stabilized vessels	Relatively simple, but burst release, dose control, and storage stability remain recurrent problems	Adjunct for acute or moderately responsive wound beds; rarely sufficient for thick constructs by itself	Extensive preclinical evidence; limited proof of durable functional perfusion
Gene-activated scaffolds	Extends local expression of pro-vascular signals within the construct	Moderate to strong—can prolong signaling beyond protein delivery	Conditional—depends on transfection efficiency, host state, and signal timing	Limited direct effect unless paired with maturation or barrier-stabilizing cues	Potentially better than single-dose proteins if sequencing is controlled, but still underdefined	More demanding; vector design, batch consistency, regulatory burden, and storage are major constraints	High-control experimental systems; possibly reconstructive settings tolerating complexity	Early preclinical evidence; scarce standardized hemocompatibility or flow data
Decellularized and micronized ECM-based systems	Provides tissue-derived ligands, matrix binding sites, and immunomodulatory cues	Moderate—supports cell attachment and biologically familiar organization	Moderate—may improve host permissiveness and graft integration	Indirect—benefit depends on retained bioactivity and pairing with vascular cells or channels	Moderate—can support immune resolution and matrix remodeling, but batch variability remains a risk	Challenging; source variability, sterilization, release testing, and storage complicate translation	Implant scaffolds, bioinks, or injectable adjuncts, depending on formulation; not one interchangeable category	Strong biologic rationale; moderate preclinical evidence; limited standardized translation data
MSCs, secretome, and EV-based trophic systems	Provides trophic, immunomodulatory, and pro-angiogenic signaling without necessarily forming vessels directly	Moderate—supports endothelial survival and stromal coordination	Indirect—may improve host responsiveness more than create ready-to-connect lumens	Limited direct effect on hemocompatibility or network architecture	Moderate—may aid resolution and remodeling, but durability is variable	MSC delivery remains variable; secretome/EV routes may improve storage and standardization	Adjunctive support strategies, especially where trophic rescue is needed	Substantial preclinical trophic evidence; weaker evidence for durable functional superiority
MVFs and SVF-containing vascular cell products	Introduces preassembled microvascular segments or multicellular vascular fractions that can connect rapidly	Strong—preserves short lumenized segments and associated support cells	Strong—among the clearest strategies for shortening inosculation	Moderate—better poised for blood entry than single-cell systems, but not immune to thrombosis or instability	Moderate to strong—depends on survival, integration, outflow, and processing quality	Difficult: donor variability, digestion workflow, storage, release criteria, and GMP adaptation are unresolved	Advanced implantable grafts where speed to connection justifies higher complexity	Strong preclinical functional rationale; limited large-animal and GMP-ready evidence
Immune-modulatory and mural-guiding strategies	Tunes macrophage timing, pericyte recruitment, and barrier maturation to convert sprouting into stable vessels	Indirect but important—shapes the early inflammatory window and sprout guidance	Moderate—productive immune tone and matrix accessibility can facilitate inosculation	Strong relevance—directly influences leakage control, barrier tightening, and regression resistance.	Strong—affects maturation, edema control, and long-term remodeling	Moderate to difficult; timing is critical, assays are complex, and indication dependence is high	Especially important in chronic, ischemic, infected, or irradiated wounds	Mechanistically persuasive; limited standardized indication-specific datasets
Bioprinted channel-based constructs and sacrificial approaches	Places cells and conduits spatially, creating predesigned transport paths and macro-to-micro organization	Moderate—offers strong spatial control, though capillary realism often remains limited.	Conditional—channels can shorten access routes, but host coupling is not automatic.	Moderate—better than static bulk gels if endothelialized; leakage and shunting remain risks	Conditional—depends on remodeling, branching logic, hemocompatibility, and outflow design.	Moderate; print speed, resolution, viable area, sterility, and workflow integration remain major trade-offs	Implant-ready custom constructs and hybrid fabrication pipelines	Strong architectural evidence; weaker evidence for durable human-scale flow competence
Perfused microfluidic and skin-on-chip platforms	Imposes controlled flow for measurement, mechanistic discovery, and product qualification under dynamic transport	Variable—can support lined, vasculogenic, or hybrid microvascular models	Not the primary aim—usually circuit-based rather than host-based	Strong for controlled flow testing; limited direct evidence of implant readiness	Strong for assay reproducibility and mechanistic benchmarking under defined conditions	Good in principle for standardization; currently limited by material variability and inter-laboratory heterogeneity	Disease modeling, transport studies, maturation, and preclinical qualification	Strong platform value for mechanism and benchmarking; not equivalent to implant efficacy evidence
Prevascularized constructs with ex vivo flow maturation	Builds and conditions the network before implantation to reduce early failure after blood entry	Strong—supports network assembly before surgery	Moderate to strong—may shorten the interval to host coupling	Strong potential—preconditioning can improve barrier behavior and reveal weak regions before implantation	Moderate to strong—if ex vivo maturation truly transfers in vivo; still unresolved at scale	Difficult; bioreactor reproducibility, sterility, release criteria, cost, and logistics are major barriers	Planned reconstructive settings more than emergency, large-area coverage	Promising preclinical evidence; substantial translational logistics gap

**Table 5:** Biomaterial design levers for perfused skin: mechanistic benefits, failure risks, and translational trade-offs [83,120,121,122,123,124].

Biomaterial Design Lever	Mechanistic Benefit for Perfused Skin	Main Risk If Mis-Tuned	The Perfusion Stage Is Most Influenced	Best-Fit Indication/Use Context	Main Translational Concern
Interconnected porosity and bulk pore size	Facilitates cell infiltration, oxygen and nutrient diffusion, endothelial invasion, and host-vessel access into the dermal compartment	Pores that are too small restrict infiltration and transport; pores that are too large can weaken mechanics, reduce cell retention, and compromise structural stability	Internal network formation; host connection	Broadly relevant across implantable dermal substitutes	No universal “optimal” pore size; swelling, fabrication method, and material chemistry can change the effective pore architecture after implantation
Hierarchical pore gradients/layer-specific architecture	Allows simultaneous support of epidermal organization at the surface and vascularized invasion deeper in the dermal region	Poor gradient design may create mismatched layers, transport dead zones, or exudate trapping	Host connection; perfusion stability	Full-thickness skin constructs and thicker dermal analogs	Difficult to manufacture reproducibly over a clinically relevant area while preserving gradient fidelity
Surface topography and microfeatures	Guides keratinocyte attachment, fibroblast organization, and endothelial cell alignment or migration at relevant interfaces	Can improve superficial adhesion without solving deeper transport or perfusion limitations if used in isolation	Internal network formation	Bilayer constructs, patterned epidermal–dermal interfaces, interface engineering	Surface effects are process-sensitive and may change after sterilization, hydration, or degradation
Stiffness and viscoelasticity	Tunes invasion permissiveness, contraction resistance, lumen support, and surgical handling	Excess stiffness suppresses invasion and sprouting; excessive softness promotes collapse, leakage, and shape loss	Host connection; flow tolerance; perfusion stability	Especially important in chronic wounds and reconstructive defects requiring mechanical persistence	Mechanical properties drift during swelling, degradation, and remodeling; cross-platform comparison remains poorly standardized
Programmed degradation/local scaffold opening	Opens space for sprouting and remodeling at the right time while preserving early structural support	Degrades too slowly and blocks invasion; degrades too quickly and causes collapse, acidic by-products, inflammatory stress, or loss of shape fidelity	Host connection; perfusion stability	Indication-specific designs, especially thick grafts and chronic wound products	Achieving reproducible spatiotemporal control of degradation is difficult to verify in release testing
Open transport channels/macro-to-micro conduits	Reduces the early diffusion bottleneck, improves convective transport, and can shorten the path between the construct core and incoming flow	Non-endothelialized or poorly integrated channels may shunt flow, leak, or mechanically weaken the scaffold	Flow initiation; oxygen rescue before stable perfusion	Thick dermal constructs, bioprinted grafts, templated scaffolds	Channel patency, host coupling, and inflow–outflow logic remain difficult to standardize at scale
Protein-loaded bioactive matrices	Provides pro-angiogenic or trophic cues that stimulate endothelial activation, sprouting, and matrix permissiveness	Burst release, short residence time, peripheral-only angiogenesis, or generation of immature/leaky vessels	Internal network formation; early host connection	Adjunctive use in acute or moderately responsive wound beds	Dose control, storage stability, and durable flow-competence data remain limited
Gene-activated matrices	Extends local expression of vascular cues and may enable better temporal control than single-dose protein delivery	Variable transfection efficiency, off-target inflammation, safety concerns, and regulatory complexity	Internal network formation; early maturation	Higher-complexity reconstructive or experimental product formats	Vector consistency, potency assays, and clinical translation pathway are more demanding than for protein-loaded scaffolds
Decellularized ECM scaffolds/dECM-based composites	Provides tissue-derived ligands, growth-factor binding sites, and immunomodulatory signals that can improve host permissiveness and remodeling	Source variability, residual immunogenic content, fast degradation, or weak mechanics if not reinforced appropriately	Host connection; perfusion stability; immune–vascular coordination	Biologically demanding dermal replacements, inflamed or remodeling-sensitive wound beds	Tissue source, decellularization chemistry, sterilization, and batch-to-batch consistency strongly affect performance
Micronized, injectable, or printable ECM-derived formulations	Offers flexible delivery, irregular defect filling, composite bioink integration, and local biologic cue enrichment	Often has limited stand-alone mechanics, rapid dilution or degradation, and dependence on a carrier matrix	Early trophic support; adjunctive network formation	Injectable adjuncts, irregular wounds, bioink systems, hybrid constructs	Rheology, sterility, storage, and preservation of bioactivity after solubilization or printing remain major hurdles

**Table 6:** Biological and cellular strategies in perfused skin engineering: when they act, what they improve, and what limits translation [64,72,97,101].

Strategy Class	The Earliest Dominant Phase of Action	Main Biological Mechanism	Strongest Contribution to the Perfusion Problem	Main Limitation If Used Alone	Translational/GMP Burden	Best-Fit Use Context	Strongest Current Evidence Base
Soluble trophic factor systems (e.g., VEGF, bFGF, PDGF)	Earliest sprouting and host activation phase	Transient pro-angiogenic and trophic stimulation of endothelial and stromal cells	Accelerates early endothelial activation and peripheral host ingrowth	Often increases vascular morphology more than durable, distributed, flow-competent perfusion; burst exposure can favor immature or leaky vessels	Low to moderate	Adjunctive support in acute or moderately responsive wound beds	Strong preclinical evidence for angiogenic stimulation; weaker evidence for durable functional perfusion
Endothelial–stromal co-culture/prevascular cellular assembly	Internal network formation before implantation	Multicellular self-assembly of endothelial cells with fibroblasts and/or mural-support cells into lumen-capable networks	Improves lumen formation, network coherence, and readiness for host inosculation	Blood-entry tolerance, long-term stability, and human-scale manufacturability remain variable.	Moderate to high	Prevascularized full-thickness constructs; ex vivo maturation systems	Strong in vitro and small-animal rationale; limited scalable translational evidence
MSC delivery	Early inflammatory and trophic phase	Paracrine immunomodulation, endothelial support, stromal rescue, and pro-repair signaling	Improves host responsiveness, dampens destructive inflammation, and supports early rescue	Engraftment is inconsistent; potency varies with source, conditioning, and delivery format.	Moderate to high	Chronic or inflamed wound beds where trophic rescue matters as much as direct vascular assembly	Broad preclinical wound-healing evidence; weaker proof of durable superiority as a stand-alone vascular strategy
MSC secretome/extracellular vesicles	Early inflammatory and trophic phase	Cell-free transfer of cytokines, growth factors, lipids, and regulatory RNAs	Offers angiogenic and immunomodulatory signaling with better storage logic than live-cell delivery	Cargo heterogeneity, dosing, release integration, and assay standardization remain unresolved	Moderate (lower live-cell burden, higher characterization burden)	Off-the-shelf adjuncts; composite scaffolds; chronic wound modulation	Rapidly growing preclinical evidence; translational standardization is still immature
Microvascular fragments (MVFs)	Immediate prevascular phase and early host connection	Preserved lumenized microvascular segments with basement membrane and associated support cells	Shortens host connection and increases the fraction of the network already structurally prepared for perfusion	Donor variability, digestion workflow, preservation, scaffold integration, and blood-entry stability remain limiting	High	Advanced implantable grafts, where rapid connection justifies higher complexity	Strong functional preclinical rationale and comparative advantage over less structured cell products; limited GMP-ready and large-animal evidence
Stromal vascular fraction (SVF)	Early trophic/pro-angiogenic support phase	Heterogeneous adipose-derived cell mixture providing paracrine angiogenic and immunomodulatory effects	Supports host responsiveness and angiogenic signaling in a relatively accessible autologous format	Lacks the preassembled lumenized segments that give MVFs a ready-to-connect advantage	Moderate to high	Autologous adjuncts and supportive vascularization strategies, rather than rapid preconnected vascular beds	Promising biologic and translational interest, but weaker direct perfusion logic than MVFs
Macrophage-directed immunomodulatory strategies	Earliest post-implant inflammatory window	Shapes the transition from inflammatory activation to resolution, influencing sprouting, remodeling, and vascular stabilization	Determines whether early angiogenesis matures into productive perfusion rather than chronic inflammatory leakage or regression	Highly timing-dependent; oversimplified M1/M2 logic can mislead design; strong indication dependence	Moderate	Diabetic, infected, ischemic, or irradiated wounds where immune dysregulation constrains vascular success	Strong mechanistic evidence; fewer standardized, indication-specific translational datasets
Pericyte/mural-guiding strategies	Transition from sprouting to maturation	Controls endothelial stabilization, basement-membrane organization, leakage resistance, and regression behavior	Improves barrier tightening, flow tolerance, and durability of remodeling	Pericytes can support or restrain angiogenesis depending on the state; phenotype control is difficult	Moderate to high	Constructs intended for durable flow competence rather than only early vessel counts	Strong mechanistic basis for maturation control; limited direct comparative datasets in skin-specific translation

**Table 7:** Comparative overview of perfused skin fabrication methods: fabrication logic, advantages, disadvantages, and translational fit.

Fabrication Method	Core Fabrication Logic	Main Perfusion Route	Key Advantages	Main Disadvantages/Limitations	Best-Fit Application Space	Key References
Porous or degradable scaffold-guided prevascularization	Natural, synthetic, or composite scaffolds are designed with interconnected pores, tunable stiffness, and degradation profiles to support endothelial/stromal invasion and vascular ingrowth.	Host-driven angiogenesis, endothelial infiltration, and progressive inosculation through the scaffold.	Technically accessible; compatible with many biomaterials; scalable over larger wound areas; can be combined with growth factors, cells, dECM, or bioactive cues; provides mechanical support for dermal replacement.	Perfusion depends heavily on host vascular competence; slow ingrowth may not rescue thick constructs in time; pore architecture and degradation may shift after swelling or implantation; vessel density does not guarantee flow competence.	Foundational platform for implantable dermal substitutes, acute wound coverage, and scaffold-based preclinical vascularization studies.	[51,61,80,83,84,85,86,87,88,89,90,91,92,93,94,95,96,111,112,118,119,120,121,122,123,124]
Scaffold-free or self-assembled prevascularized skin substitutes	Fibroblasts, endothelial cells, keratinocytes, and sometimes mural-support cells are assembled into dermo-epidermal constructs before implantation.	Preformed endothelial networks must inosculate with host vasculature after grafting.	Biologically rich; supports endothelial–stromal crosstalk; can form vessel-like networks before implantation; closer to living full-thickness skin biology than acellular scaffolds.	Manufacturing time is longer; handling of constructs can be difficult; cell-source variability is high; blood-entry tolerance, storage, and release criteria remain challenging.	Advanced living skin substitutes and planned reconstructive settings where pre-culture time is acceptable.	[30,36,56,58]
Microvascular fragment (MVF)- or SVF-assisted dermal substitute fabrication	Adipose-derived MVFs or SVFs are incorporated into dermal matrices to introduce vascular/stromal cellular units.	MVFs provide partially preserved, lumenized vascular segments that can rapidly connect to host vessels; SVFs mainly provide trophic and pro-angiogenic support.	MVFs offer one of the clearest strategies for accelerating early host connection, preserving multicellular vascular units, and improving vascularization, lymphangiogenesis, integration, oxygenation, and earlier grafting.	Donor variability; enzymatic digestion and processing burden; difficult GMP standardization; limited storage window; SVF lacks the preassembled lumenized structure of MVFs; blood-entry stability still requires validation.	Advanced implantable grafts in which shortening the ischemic interval justifies greater processing complexity.	[72,73,74,75,76,125,126,127,128]
Conventional extrusion/inkjet/layer-by-layer skin bioprinting	Cells and bioinks are deposited layer by layer to generate dermal and epidermal compartments, optionally including endothelial or perivascular cells.	Primarily self-assembled endothelial networks, printed cell patterns, and later host inosculation; perfusion may be indirect unless channels are incorporated.	Enables spatial organization of keratinocytes, fibroblasts, endothelial cells, pericytes, and matrix components; supports patient- or defect-specific geometry; suitable for multilayered skin construction.	Resolution–speed–viability trade-off; true capillary-scale channels are difficult to print directly; printed geometry can change due to contraction and remodeling; perfusion may remain incomplete without conduit design.	Patient-specific graft prototypes, vascularized skin analogs, and in vitro or preclinical bioprinted constructs.	[37,38,39,51,92,93,94,108,109]
Sacrificial or fugitive-ink channel templating	A removable material such as Pluronic F127, gelatin, agarose, alginate, or another fugitive ink is printed or molded inside a matrix and later removed to leave hollow channels.	Hollow channels can be perfused directly and then endothelialized; channels may serve as macro-scale conduits for later capillary self-assembly.	Creates continuous perfusable paths; improves convective transport; useful for reducing diffusion limitations in thick constructs; compatible with hybrid bioprinting workflows.	Channel diameters are often larger than native capillaries; endothelialization can be uneven; risks of leakage, shunting, collapse, or poor inflow–outflow balance; channel presence alone does not ensure host integration.	Thick constructs needing early convective transport, perfusable in vitro models, and hybrid implant-development platforms.	[40,41,42,43,44,45,94,133]
Light-based printing, DLP, stereolithography, and projection microfabrication	Photocrosslinkable bioinks are patterned with light to generate high-fidelity microfeatures, channels, or multilayered structures.	Defined channels, patterned compartments, or microarchitectures support imposed flow or subsequent endothelialization.	High spatial resolution; reproducible patterning; strong potential for standardized microchannel arrays and model platforms; useful for precise architecture–transport studies.	Restricted bioink palette; photoinitiator and light-exposure cytotoxicity concerns; limited suitability for large, soft, cell-rich implantable grafts; scaling to clinically relevant areas is difficult.	High-resolution in vitro models, microstructured dermal platforms, and standardized qualification systems, rather than immediate large-area grafts.	[37,38,39,51,108,109]
Endothelial-lined microfluidic skin-on-chip systems	Predefined microchannels or compartments are fabricated in a chip and lined with endothelial cells alongside dermal and epidermal compartments.	Externally imposed microfluidic perfusion with defined flow rate, shear stress, and nutrient exchange.	Excellent control over flow, shear, permeability, barrier function, and real-time readouts; strong platform for disease modeling, drug testing, toxicology, and mechanistic studies.	Not an implantable graft; simplified vascular geometry; device material effects and medium formulation can alter biology; throughput and inter-laboratory standardization remain issues.	Mechanistic discovery, barrier/transport studies, toxicology, inflammation modeling, and preclinical qualification.	[31,32,33,34,35,46,47,48,49,50,57,59,62,63,77,78]
Vasculogenic microfluidic self-assembled networks	Endothelial and stromal cells self-organize into capillary-like microvascular networks inside hydrogel compartments under microfluidic control.	Self-assembled microvessels connect to microfluidic reservoirs or channels and can support tracer or medium perfusion.	More biologically realistic capillary organization than simple straight channels; useful for studying angiogenesis, permeability, sprouting, and cell–cell crosstalk.	Network topology is variable; reproducibility is lower than that of predefined channels; scalability is limited; and direct assessment of connection to the host macrovasculature is not available.	Human-relevant microvascular biology, comparative testing of biomaterials or cells, and mechanistic perfusion studies.	[46,47,48,49,50,57,59,62,63,77,78]
Hybrid patterned-conduit plus self-assembled capillary systems	Larger printed or molded conduits are combined with endothelial/stromal self-assembly to bridge macroscale transport pathways and capillary-like networks.	Patterned channels provide primary flow paths, while surrounding self-assembled microvessels provide microvascular distribution.	Strong conceptual match to hierarchical vascular architecture; combines controllability with biological realism; may reduce shunting if macro-to-micro integration is achieved.	Multi-step fabrication; difficult sterility and reproducibility; unresolved venous outflow and thrombosis risks; capillary–conduit integration may be incomplete or unstable.	One of the most promising routes for clinically relevant perfusion design and advanced qualification platforms.	[42,43,44,45,94,133]
Ex vivo perfusion conditioning/on-chip vascular maturation	Prevascularized constructs are exposed to controlled flow before implantation or used under flow as a release-testing or maturation step.	Imposed flow conditions mature endothelial barriers, test patency, and reveal weak regions before blood exposure or implantation.	Improves endothelial readiness; enables stress testing of leakage and flow tolerance; supports quality-control logic; may help bridge the gap between in vitro maturation and in vivo performance.	Adds time, cost, sterility burden, bioreactor complexity, and release-test requirements; may be unsuitable for urgent large-area burns; does not itself solve recipient macrovascular coupling.	Planned reconstructive grafts, high-value living constructs, and preclinical qualification workflows.	[30,31,48,49,50,57,59,62,63,77,78,114,115,116,117,133]

**Table 8:** Minimum translational dataset for vascularized skin constructs [50,114,116,134].

Translational Domain	Minimum Required Evidence	Representative Assay/Study Format	Minimum Evidence Level Before Serious Translation	Why Omission Is Dangerous
Time to functional perfusion	Distributed perfusion within a clinically relevant time window, not just peripheral filling or occasional blood entry	Intravital imaging, fluorescent tracer or RBC transit mapping, depth-resolved perfusion assessment	Dynamic in vitro evidence plus in vivo confirmation in an indication-relevant wound model	Favorable vascular morphology can mask persistent core ischemia
Patency persistence during remodeling	The network remains open and perfused beyond transient early filling	Longitudinal perfusion imaging, repeated tracer studies, functional–histologic correlation	Follow-up beyond the initial connection window	Early “success” may disappear before durable wound rescue is achieved
Hemocompatibility/thrombogenicity	No rapid clotting, platelet/fibrin accumulation, or catastrophic occlusion at blood entry	Whole-blood flow loop, ex vivo shunt, dynamic perfusion clot assays, and clot burden scoring	Pre-implant dynamic blood-contact testing plus post-implant verification	A construct can fail at the very first encounter with blood, even if the vascular geometry looks excellent
Leakage control/endothelial barrier behavior	Acceptable permeability and endothelial retention under flow	Dextran leakage assays, permeability testing, junctional staining under flow, live barrier imaging	Controlled flow testing and early post-implant assessment	Excess leakage promotes edema, poor oxygen efficiency, and unstable integration
Inflow–outflow balance/venous drainage	Perfusion is distributed rather than shunted; no major congestion, pooling, or one-sided inflow	Perfusion maps, outflow collection, edema scoring, pressure-drop analysis, and outflow-side imaging, where possible	Functional evidence in scaled constructs or surgically relevant settings	A construct may appear perfused while most of the tissue remains underserved
Full-thickness tissue rescue	Oxygen/nutrient rescue and viability are maintained across the intended construct thickness	Oxygen probes, hypoxia mapping, viability gradients, depth-resolved histology, metabolic readouts	Thickness-matched in vitro and in vivo evidence	Thin-model success can dramatically overpredict performance in clinically thicker grafts
Barrier recovery at the skin level	Downstream skin function improves in a way consistent with the platform’s purpose	TEER/TEWL, epidermal differentiation markers, barrier recovery assays, wound closure metrics	Construct-level evidence linked to vascular performance	Perfusion may be present without translating into meaningful skin function
Surgical handling and fixation	The construct can be transferred, trimmed, secured, and conformed to irregular wound beds without tearing or collapsing	Simulated OR handling, suture/staple tests, drape/contour assessment, implantation workflow testing	Bench handling plus implantation workflow evidence	Biologically sophisticated products can fail in routine surgical use
Infection resilience/wound-bed compatibility	The construct retains performance in contaminated, inflamed, ischemic, or otherwise compromised beds relevant to its intended indication	Infected/ischemic/chronic wound models, microbial challenge, cytokine-stress assays	Indication-matched preclinical evidence	Data from clean, healthy beds can substantially overestimate clinical performance
Manufacturing consistency and potency/release framework	Batch reproducibility and release assays are linked to the intended mechanism of action	QC panel, potency assay, in-process controls, comparability testing after process change	Preclinical development package before scale-up claims	Without this, efficacy cannot be reproduced or credibly translated
Storage, logistics, and deployment window	Acceptable interval between manufacture and use for the target clinical scenario	Shelf-life testing, transport stress studies, thaw/recovery testing, and ready-to-use workflow evaluation	Matched to acute versus elective clinical workflow	A promising construct may still be unusable in real practice
Comparative value versus standard of care	The construct is benchmarked against the therapy it aims to replace or augment	Head-to-head comparison versus split-thickness grafts, dermal substitutes, or chronic wound standards	Comparative evidence before claims of superiority or clinical necessity	Translation remains abstract unless benefit is shown in context

## Data Availability

No new data were created or analyzed in this study. Data sharing is not applicable.
